# Specifically blocking αvβ8-mediated TGF-β signaling to reverse immunosuppression by modulating macrophage polarization

**DOI:** 10.1186/s13046-024-03250-1

**Published:** 2025-01-02

**Authors:** Cuicui Guo, Hui Sun, Yulei Du, Xiaodong Dai, Yu Pang, Zhen Han, Xinhui Xiong, Shaowei Li, Junhua Zhang, Qingbing Zheng, Xun Gui

**Affiliations:** 1 Mabwell (Shanghai) Bioscience Co., Ltd, Shanghai, 201210 China; 2 State Key Laboratory of Vaccines for Infectious Diseases, National Institute of Diagnostics and Vaccine Development in Infectious Diseases, School of Public Health, Xiamen University, Xiamen, China; 3 Nanjing Novoacine Biotechnology Co., Ltd, Nanjing, 210032 China; 4 State Key Laboratory of Molecular Vaccinology and Molecular Diagnostics, Xiang An Biomedicine Laboratory, Xiamen University, Xiamen, China; 5 Department of Radiation Oncology, Fudan University Shanghai Cancer Center, Shanghai, 200032 China

**Keywords:** TGF-β, Tumor microenvironment, Macrophage polarization, Immune cell infiltration

## Abstract

**Background:**

Targeting the TGF-β pathway in tumor therapy has proven challenging due to the highly context-dependent functions of TGF-β. Integrin αvβ8, a pivotal activator of TGF-β, has been implicated in TGF-β signaling within tumors, as demonstrated by the significant anti-tumor effects of anti-αvβ8 antibodies. Nevertheless, the expression profile of αvβ8 remains a subject of debate, and the precise mechanisms underlying the anti-tumor effects of anti-αvβ8 antibodies are not yet fully elucidated.

**Methods:**

We utilized single-cell RNA sequencing to assess αvβ8 expression across various human tumors. An anti-αvβ8 antibody was developed and characterized for its binding and blocking properties in vitro. Cryo-EM single-particle analysis was employed to study the detailed interaction between αvβ8 and the antibody Fab fragment. The anti-tumor efficacy of the antibody was evaluated in syngeneic mouse models with varying levels of αvβ8 expression, both as a monotherapy and in combination with PD-1 antibodies. Human PBMCs were isolated to investigate αvβ8 expression in myeloid cells, and macrophages were exposed to the antibody to study its impact on macrophage polarization. Pharmacokinetic studies of the αvβ8 antibody were conducted in cynomolgus monkeys.

**Results:**

Integrin αvβ8 is notably expressed in certain tumor types and tumor-infiltrating macrophages. The specific αvβ8 antibody 130H2 demonstrated high affinity, specificity, and blocking potency in vitro. Cryo-EM analysis further revealed that 130H2 interacts exclusively with the β8 subunit, without binding to the αv subunit. In vivo studies showed that this antibody significantly inhibited tumor growth and alleviated immunosuppression by promoting immune cell infiltration. Furthermore, combining the antibody with PD-1 inhibition produced a synergistic anti-tumor effect. In human PBMCs, monocytes exhibited high αvβ8 expression, and the antibody directly modulated macrophage polarization. Tumors with elevated αvβ8 expression were particularly responsive to 130H2 treatment. Additionally, favorable pharmacokinetic properties were observed in cynomolgus monkeys.

**Conclusions:**

In summary, integrin αvβ8 is highly expressed in certain tumors and tumor-infiltrating macrophages. Targeting αvβ8 with a blocking antibody significantly inhibits tumor growth by modulating macrophage polarization and enhancing immune cell infiltration. Combining αvβ8 targeting with PD-1 treatment markedly increases the sensitivity of immune-excluded tumors. These results support further clinical evaluation of αvβ8 antibodies.

**Graphical abstract:**

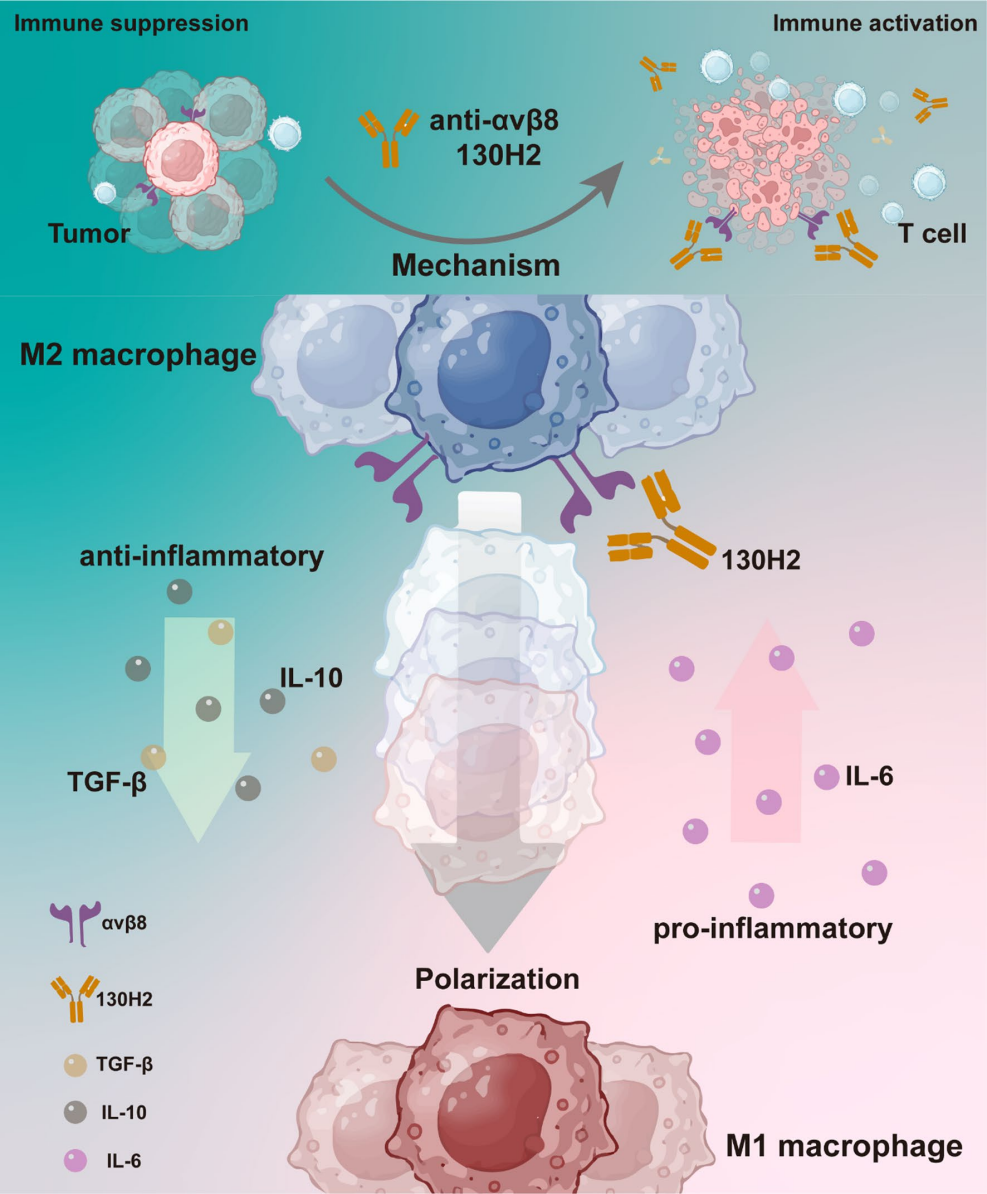

**Supplementary Information:**

The online version contains supplementary material available at 10.1186/s13046-024-03250-1.

## Introduction

The role of Transforming Growth Factor β (TGF-β) is pivotal in regulating development and maintaining immune homeostasis, particularly in the tumor microenvironment (TME). TGF-β is abundantly present in the TME and enforces immune suppression through various mechanisms, including dampening inflammatory responses within the TME [1], hindering Th1 helper and cytotoxic T cell reactions [2, 3], promoting T regulatory phenotype [4], and suppressing Natural Killer cells (NK) [5], as well as regulating macrophage activity [6]. Consequently, targeting TGF-β pathway to unleash the immune system against tumors represents a promising strategy for cancer therapy.

Three highly homologous TGFβ isoforms, TGFβ1, TGFβ2, and TGFβ3, exist in mammals. Among these, TGF-β1 is the predominant isoform and the most relevant member of the family in terms of immune regulation. All three forms are synthesized as prohormones and exist in a latent form (L-TGF- βs) by noncovalent association with the latency-associated peptide (LAP) and must be activated to exert biological functions [7, 8]. The release of active TGF-β from latent complexes is a tightly regulated process involving both enzymatic and non-enzymatic activities present in the extracellular space. Previous studies have indicated that integrins, specifically αvβ6 and αvβ8, can bind the RGD site in L-TGF-β1 and L-TGF-β3, facilitating the release of active TGF-β homodimers from the latent complexes [7]. In contrast, L-TGF-β2 lacks a typical RGD domain and may be activated by other, yet unknown, mechanisms [9]. Both αvβ6 and αvβ8 are members of integrin family, which are heterodimers composed of non-covalently associated α and β subunits.

The importance of TGF-β activation by integrins has been demonstrated in mice. Specifically, mice with a mutation in the RGD domain that abrogates integrin binding recapitulate the phenotype of TGF-β1 null mice [10]. Mice with silenced β8 encoding gene *ITGB8* die either at midgestation or shortly after birth, and conditional knockout of *ITGB8* results in severe inflammatory bowel disease. In contrast, mice lacking β6 encoding gene *ITGB6* exhibit a very mild phenotype. The expression of αvβ8 is distinct among the αv integrins, particularly in its expression by immune cells such as dendritic cells (DCs) [11]. It has been identified as a contextual activator of TGF-β, playing a crucial role in regulating active immune responses. The expression of αvβ8 by DCs mediates TGF-β production during T cell activation, significantly influencing the differentiation and development of regulatory T cells (Tregs) and Th17 cells, while concurrently inhibiting Th1 cell differentiation during immune responses [12, 13]. In murine models, the conditional knockout of *ITGB8* on leukocytes leads to the development of severe inflammatory bowel disease and age-associated autoimmune disorders [11]. These findings collectively demonstrate that integrin αvβ8 is a key activator of latent TGF-β1 and serves as the main regulator of TGF-β in immune cells.

Efforts to target the TGF-β pathway have been ongoing for years but have been hampered by systemic toxicity [14] and limited efficacy due to the pleiotropic and highly context-dependent functions of TGF-β [15, 16]. Strategies that restrict TGF-β inhibition to specific biological contexts, particularly within the suppressive TME, could offer improved safety and therapeutic advantages over broad TGF-β inhibition. In our study, we employed single-cell RNA sequencing (scRNA-seq) to analyze various human tumor types, revealing that the integrin αvβ8 is expressed in both tumor cells and tumor-infiltrating macrophages. Leveraging the specific expression of integrin αvβ8, we investigated its role in the activation of TGF-β, focusing on its spatial and temporal functions. Blocking integrin αvβ8 resulted in significant tumor inhibition across multiple tumor models. Treatment with a monoclonal anti-αvβ8 antibody led to a marked increase in tumor-infiltrating immune cells, including CD8 + T cells, CD4 + T cells, DCs, and NK cells. Furthermore, in vitro incubation with the anti-αvβ8 antibody induced monocyte polarization towards an M1 macrophage phenotype. In conclusion, anti-αvβ8 antibody therapy exerts substantial tumor-inhibitory effects by promoting immune cell infiltration and modulating macrophage polarization towards a more pro-inflammatory M1 phenotype.

## Materials and methods

### Single-cell RNA-seq data source

Ten male patients clinically diagnosed with KIRC were enrolled in this study at Fudan University Shanghai Cancer Center. Their ages ranged from 50 to 75. All of patients were newly diagnosed with KIRC and none of the patients had received therapy (supplemental Table 1). Written informed consent was provided by all patients. This study was approved by the Research and Ethical Committee of Fudan University Shanghai Cancer Center and complied with all relevant ethical regulations. Tumor cells of KIRC patient were counted and loaded onto the Chromium Controller (10X Genomics) for a target recovery of 81718 single cells. Samples were processed for scRNA-seq library preparation with 10X Chromium 5’ kit, following the manufacturer’s protocol and sequenced on an Illumina platform. The raw data were processed using Cell Ranger software (v6.1.2). The reads were aligned to human genome GRCh38, and a gene count matrix was generated for each sample. The raw count data were then loaded into Seurat package (v4.3.0) for quality control, filtering, normalization, UMAP visualization, and clustering. Cells with fewer than 200 genes, more than 10,000 genes, or with mitochondrial gene content greater than 25% were filtered out. Data from 10 patients were analyzed after Harmony integration. Cell clusters were identified using classical gene markers, and the top three genes in the dot plot are shown in supplemental Fig. 1A. HNSCC scRNA-seq data were obtained from the GEO dataset-GSE234933 [17]. The top three genes in the dot plot are shown in supplemental Fig. 1B.

For the investigation of the expression of αvβ8 on Treg cells, we isolated Treg cell subsets from KIRC and HNSCC single-cell datasets using IL2RA, FOXP3, IL32, LTB. Additionally, in supplemental Fig. 6, we explored the expression of αvβ8 on Tregs in six other types of cancer, including esophageal cancer (ESCA; GSE160269) [18], liver hepatocellular carcinoma (LIHC; GSE166635) [19], lung squamous cell carcinoma (LSCC; GSE150321) [20], nasopharyngeal cancer (NPC; GSE162025) [21], non-small cell lung cancer (NSCLC; EMTAB6149) [22], and oral squamous cell carcinoma (OSCC; GSE172577) [23]. Cell annotation refers to the original papers and TISCH2 [24].

### Cells

HEK293-6E cells were procured from the American Type Culture Collection (ATCC) and cultured in FreeStyle™ F17 medium (Invitrogen, USA). Human glioblastoma U251MG cells, ovarian carcinoma OVCAR3 cells, murine mammary carcinoma EMT6, murine lung adenocarcinoma LA795, and murine colorectal carcinoma CT26 cells were obtained from CoBioer Biotechnology Co., Ltd. (Nanjing, China). U251MG cells were maintained in Minimum Essential Medium (MEM; Gibco, USA) supplemented with 10% fetal bovine serum (FBS; Gibco), 1% non-essential amino acids (Gibco), and 1 mM sodium pyruvate (Gibco). OVCAR3 cells were cultured in RPMI-1640 medium (Gibco) containing 20% FBS and 10 µg/mL insulin (Beyotime Biotechnology, China). EMT6 cells were fed with Waymouth’s 752/1 medium complete medium (CoBioer). LA795 and CT26 cells were cultured in RPMI-1640 medium with 10% FBS. LA795 cells overexpressing human αvβ8 were engineered following standard protocols. Frozen peripheral blood mononuclear cells (PBMCs) were purchased from Shanghai Hycells Biotechnology Co., Ltd. (Shanghai, China). These cells were isolated from healthy male Asian donors aged 20 to 30 years through ethically approved procedures.

### Animals

Female BALB/c mice (six to eight weeks old) and male Kunming mice were purchased from Yicon BioMedical Technology Inc. (Beijing, China).

### Blocking activity determination

For the protein-based blocking assay, αvβ8 protein was coated onto a white opaque 96-well plate at a concentration of 5 µg/mL. Human HEK293T/GARP/latent-TGFβ1/TGFβRII-luciferase cells were used as effector cells. Serial dilutions of antibodies were pre-incubated with effector cells at 37 ℃, 5% CO_2_ for 1 h. Subsequently, effector cells were collected and added to the corresponding wells at a density of 4 × 10^4^ cells per well. After 5 h of incubation, 100 µL of Bio-Lite detection substrate (Vazyme, China) was added to detect luciferase activity. Relative luminescence units (RLU) were measured using a SpectraMax M5e Microplate Reader (Molecular Devices, USA).

For the cell-based blocking assay, human HEK293T/GARP/latent-TGFβ1/TGFβRII-luciferase cells and U251MG cells were used as effector and target cells, respectively. Target cells were collected and seeded into a white opaque 96-well plate at a density of 2 × 10^4^ cells per well. Serial dilutions of antibodies were pre-incubated with the target cells at 37 °C and 5% CO_2_ for 1 h. Subsequently, effector cells were collected and added to the corresponding wells at a ratio of 2:1 (effector to target cells). After 5 h of incubation, 100 µL of Bio-Lite detection substrate was added and relative luminescence units (RLU) were measured using a SpectraMax M5e Microplate Reader. Data for both experiments were derived from three independent experiments, each performed in duplicate.

### Cryo-EM sample and data collection

Aliquots (3 µL) of 2 mg/mL mixtures of the human integrin αvβ8 (ACROBiosystems) in complex with excess Fab fragments of mAb 130H2 were prepared. These samples were incubated and then loaded onto glow-discharged (60 s at 20 mA) holey carbon Quantifoil grids (R1.2/1.3, 200 mesh, Quantifoil Micro Tools) using a Vitrobot Mark IV (ThermoFisher Scientific) at 100% humidity and 4 °C. Data acquisition was conducted using the smart EPU software on a Titan Krios G4 transmission electron microscope (ThermoFisher Scientific) operated at 300 kV and equipped with a Gatan K3 direct detector and a BioContinuum HD Imaging Filter functioning in zero-loss mode with a slit width of 20 eV. Images were recorded in the 48-frame movie mode at a nominal magnification of 130,000 × in super-resolution mode, yielding a pixel size of 0.325 Å. The total electron dose was set to 48 e-/Å2 with an exposure time of 1.33 s.

### Image processing and 3D reconstruction

Drift and beam-induced motion correction were performed with MotionCor2 [25] to produce a micrograph from each movie. Contrast transfer function (CTF) fitting and phase-shift estimation were conducted with Gctf [26]. Micrographs with astigmatism, obvious drift, or contamination were discarded before reconstruction. The subsequent reconstruction procedures were performed using Cryosparc V4 [27]. Briefly, particles were automatically picked using the “Template picker” tool. Several rounds of reference-free 2D classifications were conducted, and selected good particles were subjected to ab-initio reconstruction, heterogeneous refinement, and final non-uniform refinement. The resolution of all density maps was determined by the gold-standard Fourier shell correlation curve with a cutoff of 0.143 [28]. Local map resolution was estimated with ResMap [29].

### Atomic model building, refinement, and 3D visualization

The initial model of the fragment variable of 130H2 was generated from homology modeling by Accelrys Discovery Studio software (available from: URL: https://www.3dsbiovia.com). The structure of αvβ8 from the previously reported structure (PDB ID: 6UJA [30]) was served as the initial model. We initially fitted the template into the corresponding final cryo-EM maps using Chimera [31], and further corrected and adjusted them manually by real-space refinement in Coot [32]. The resulting models were then refined with phenix.real_space_refine in PHENIX [33]. These iterative processes continued until problematic regions, Ramachandran outliers, and poor rotamers were either corrected or moved to favored regions. The final atomic models were validated using Molprobity [34, 35]. All figures were generated with Chimera or ChimeraX [36, 37].

### In vivo efficacy studies in syngeneic tumor models

BALB/c mice were subcutaneously inoculated with breast cancer EMT6 cells (1 × 10^5^ in 100 µL per mouse). When the tumor volume reached approximately 100 mm^3^, the mice were divided into two groups of fourteen mice each and intraperitoneally administered either 10 mg/kg isotype control antibody or 10 mg/kg 130H2 every four days. Tumor volume and body weight were measured three times a week. Mice were sacrificed on day 12 and day 17 post-inoculation, and tumors were resected for tumor-infiltrating lymphocyte analysis. The cell populations of CD4 T cells, CD8 T cells, M1 macrophages, M2 macrophages, MDSCs, DC and NK cells were distinguished using markers including CD45、CD11b、CD3、CD4、CD8、F4/80、Gr-1、CD335、MHC II、CD206、CD11c.

EMT6 tumor models were used to investigate the synergistic effects of 130H2 in combination with a PD-1 antibody. EMT6-bearing mice were randomly assigned into four groups of six mice each when the average tumor size was approximately 100 mm^3^. The groups received the following treatments: isotype-hIgG1 (10 mg/kg) plus isotype-rat IgG2a (10 mg/kg), 130H2 (10 mg/kg) plus isotype-rat IgG2a (10 mg/kg), isotype-hIgG1 (10 mg/kg) plus mouse PD-1 antibody (clone RMP1-14, 10 mg/kg), and 130H2 (10 mg/kg) plus RMP1-14 (10 mg/kg). 130H2 and isotype-hIgG1 were administered once a week for two weeks on day 5 and 12, while RMP1-14 and isotype-rat IgG2a were administered every four days, on day 5, 9, and 13, totaling three administrations. Tumor volume and body weight were measured and recorded three times a week. Mice were sacrificed on day 15 post-inoculation, and tumors were resected for tumor-infiltrating lymphocyte analysis.

BALB/c mice were subcutaneously inoculated with colorectal cancer CT26 cells (1 × 10^5^ in 100 µL per mouse). When the tumor volume reached approximately 100 mm^3^, the mice were divided into two groups (*n* = 7). They were treated with 10 mg/kg isotype control antibody, or 10 mg/kg 130H2 every five days. Tumor volume and body weight were measured three times a week. Mice were sacrificed on day 15 after tumor inoculation.

### In vivo efficacy studies in αvβ8 overexpressed tumor models

LA795 lung adenocarcinoma cells and human αvβ8 over-expressed LA795 cells were utilized to establish xenograft models for evaluating the in vivo efficacy of 130H2. Specifically, 100 µL of LA795 cells or LA795-αvβ8 cells, corresponding to 1 × 10^7^ cells per mouse, were subcutaneously transplanted into the right flanks of Kunming mice. Once the tumor volume reached approximately 100 mm^3^, the mice were randomized into two groups of seven each. These groups received intravenous treatments of either 10 mg/kg isotype control antibody or 130H2, administered once every four days for a total of three doses. Body weight and tumor size were measured biweekly.

In a separate experiment, twenty-eight LA795-αvβ8-bearing mice were randomized into four groups: 5 mg/kg isotype control, 5 mg/kg isotype control-LALA, 5 mg/kg 130H2, and 5 mg/kg 130H2-LALA. Antibodies were administered via tail vein injections every four days for a total of three doses. Tumor volume and body weight were recorded triweekly.

### Human PBMC derived M1 and M2-like macrophages and DCs preparation

CD14 + monocytes were isolated from frozen PBMCs using CD14 microbeads (Miltenyi Biotec, USA). The isolated monocytes were then suspended in RPMI 1640 medium supplemented with 10% FBS at a density of 1 × 10^6^ cells/mL. The monocytes were divided into four groups, with three groups being supplemented with 50 ng/mL macrophage colony-stimulating factor (M-CSF, Peprotech, USA), 50 ng/mL granulocyte-macrophage colony-stimulating factor (GM-CSF, Peprotech), or GM-CSF plus IL-4 (Peprotech) to generate M2 and M1-like macrophages and DCs, respectively. After a 7-day incubation period at 37 °C, the cells were harvested and characterized by CD163 and CD86 markers. Then, macrophages and DCs were stained to detect the expression of αvβ8 integrin. Results were derived from three independent experiments using samples from six donors.

### Regulatory T cells preparation and αvβ8 detection

Human regulatory T cells (CD4 + CD25 + CD127low regulatory T cells) were isolated from frozen PBMCs (five donors) using EasySep™ human regulatory T cell isolation kit (Stemcell Technologies, Canada). The isolated cells were resuspended in RPMI-1640 medium at a density of 0.5 × 10^6^/mL, then supplemented with CD3/CD28 Dynabeads (Gibco) and 50 ng/mL IL-2 (Peprotech, USA) to activate and expand the cells.

After three days, cells were harvested to assess αvβ8 expression as described below. The cells were washed three times with staining buffer (PBS containing 1% FBS) and resuspended at a density of 2 × 10^6^/mL. For surface marker staining, 2 µL FITC-anti-CD4 (BioLegend), 2 µL PE-Cy7-anti-CD25 (BioLegend), and 100 nM APC-130H2 were added to 100 µL of the cell suspension. Single-stained controls for each fluorochrome and unstained controls were prepared simultaneously. Following a 1-hour incubation at 4 °C in the dark, the cells were washed three times with staining buffer. Next, the cells were resuspended in 200 µL of 1× Fixation/Permeabilization buffer and incubated at 4 °C in the dark for 1 h. After the supernatant was discarded, the cells were washed and resuspended in 1× Permeabilization buffer. To stain intracellular markers, 2 µL PE-anti-FoxP3 (BioLegend) was added to the cell suspension, followed by a 30-minute incubation at 4 °C in the dark. The cells were washed with Permeabilization buffer and resuspended in staining buffer for immediate analysis using a CytoFLEX flow cytometer (Beckman Coulter).

### Characterization of macrophage polarization

CD14 + monocytes derived from PBMC (six donors) were stimulated with 50 ng/mL M-CSF to differentiate into M2-like macrophages. After four days, 10 µg/mL of 130H2, TGF-βRII, and an isotype control antibody were added to the cultures, respectively, and co-incubated for an additional three days. The supernatants were then collected to measure the concentrations of interleukin-6 (IL-6) and interleukin-10 (IL-10) using homogeneous time-resolved fluorescence (HTRF) detection kits (PerkinElmer, Inc., USA). The experiments were performed in duplicate across three independent trials.

### Single-dose pharmacokinetics study

One male and one female cynomolgus monkeys were recruited into a single-dose pharmacokinetics study. They were administrated 10 mg/kg of 130H2 via a single 1-hour intravenous infusion. Blood samples without anticoagulation were collected at the following time points: 0 h (+ 1 min), 2 h (± 5 min), 8 h (± 10 min), 24 h (± 30 min), 48 h (± 30 min), 72 h (± 30 min), 96 h (± 30 min), 120 h (± 30 min), 168 h (± 30 min), 336 h (± 60 min), 504 h (± 60 min). The concentration of the antibody in serum was quantified using an enzyme-linked immunosorbent assay (ELISA).

### Statistical analysis

Tumor volume was calculated according to the formula: tumor volume (mm^3^) = length × (width)^2^ × 0.5. Tumor growth inhibition (TGI) was figured out using the following formula: TGI_TV_ (%) = (1 – TVt/ TVc) × 100% or TGI_TW_ (%) = (1 – TWt/ TWc) × 100%, where TVt, TWt was the average tumor volume or weight of treated group and TVc, TWc was the average tumor volume or weight of isotype control group. GraphPad Prism 9.0.0 software was used for graph creation and analysis of intergroup differences. Intergroup differences for all immunological cells were analyzed using Student’s t-test. Specifically, intergroup differences in Figs. 4B–J and 5C–E were evaluated using the t-test. For Fig. 4K, two-way ANOVA with Tukey’s multiple comparisons was applied. One-way ANOVA with Dunnett’s multiple comparisons test was used for Fig. 4L–O. In addition, the Wilcoxon test was employed for the analysis in Fig. 6A. For Fig. 6C, a two-way ANOVA with multiple comparisons was conducted. *P* < 0.05, *P* < 0.01, *P* < 0.001, *P* < 0.0001 was noted as *, **, ***, **** respectively, and *P* < 0.05 was considered statistically significant.

## Results

### scRNA-seq of KIRC and HNSCC reveals high expression of αvβ8 in tumors and tumor-infiltrating macrophages

**Fig. 1:**
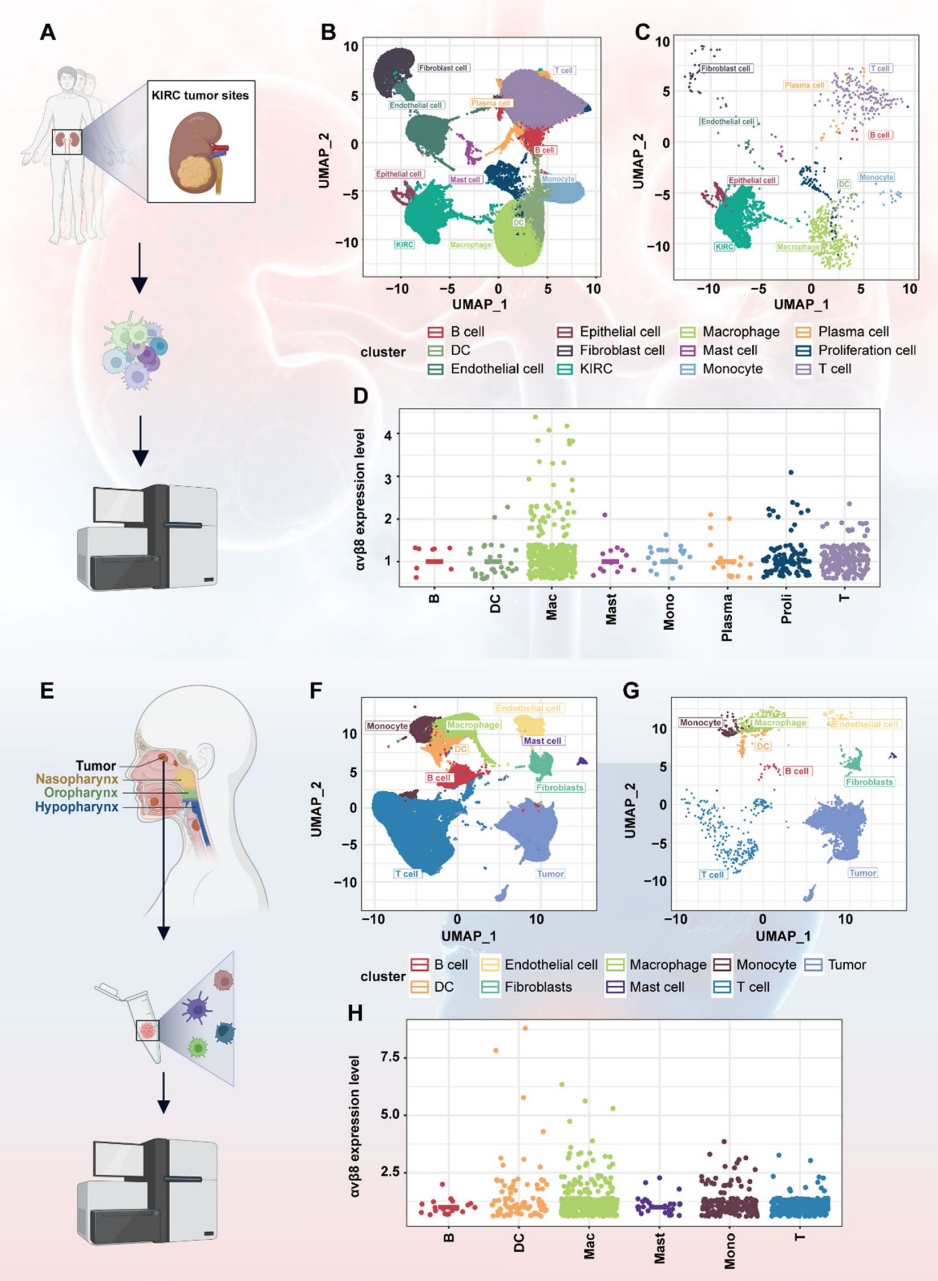
Integrin αvβ8 expression in KIRC and HNSCC as revealed by scRNA-seq analysis. **(A)** Ten freshly collected primary KIRC tissues were subjected to scRNA-seq analysis. **(B)** UMAP lots of KIRC patient cells, colored to depict the 12 identified clusters. **(C)** Distribution of *ITGB8*-expressing cells within each cluster of KIRC. **(D)** Point plot illustrating the distribution of *ITGB8*-expressing cells across various immune cell types in KIRC. **(E)** Analysis of 52 HNSCC tumor samples from individual patients as referenced in the literature. **(F)** UMAP plots of HNSCC patient cells, colored to show the 9 identified clusters. **(G)** Distribution of *ITGB8*-expressing cells within each cluster of HNSCC. (**H)** Point plot illustrating the distribution of *ITGB8*-expressing cells across various types of immune cells of HNSCC

To investigate the expression of integrin αvβ8 in tumor cells and immune cells infiltrating the TME, we selected kidney renal clear cell carcinoma (KIRC) for scRNA-seq. Ten freshly collected primary KIRC tissues were analyzed using the 10X Genomics platform (Fig. 1A). Unsupervised clustering with the GraphClust algorithm, followed by visualization in Uniform Manifold Approximation and Projection (UMAP) space, revealed diverse cellular identities based on known canonical cell markers in primary KIRC. We identified 12 distinct cell types from a total of 81,718 cells (Fig. 1B). The markers for the cell clusters are presented in supplemental Fig. 1A. As expected, *ITGB8* was predominantly expressed in epithelial cells or epithelial cell-derived tumors (Fig. 1C). The abundance of *ITGB8* in tumor cells was significantly higher than in immune cells, prompting a re-analysis of tumor-infiltrating immune cells with the exclusion of tumor cells. To our surprise, among immune cells, *ITGB8* exhibited the highest expression in macrophages (Fig. 1D).

To validate this result, we utilized sequencing data from an external study to analyze the expression of αvβ8. The referenced study [17] included a total of 52 head and neck cancer samples, each derived from individual patients (Fig. 1E). After quality control and filtering, as described by Bill et al., nine cell clusters were identified (Fig. 1F). The markers for the cell clusters are detailed in supplemental Fig. 1B. *ITGB8* was highly expressed in tumor cells (Fig. 1G). After removing tumor cells, *ITGB8* was predominantly expressed in myeloid cells, particularly macrophages (Fig. 1H). These data collectively verify that αvβ8 encoding gene is most highly expressed by tumor cells. Additionally, among tumor-infiltrating immune cells, macrophages might play a significant role in the occurrence of tumors related to the αvβ8-TGF-β signaling pathway.

### Anti-αvβ8 antibody exhibits high binding affinity and potent TGF-β blocking activity

**Fig. 2:**
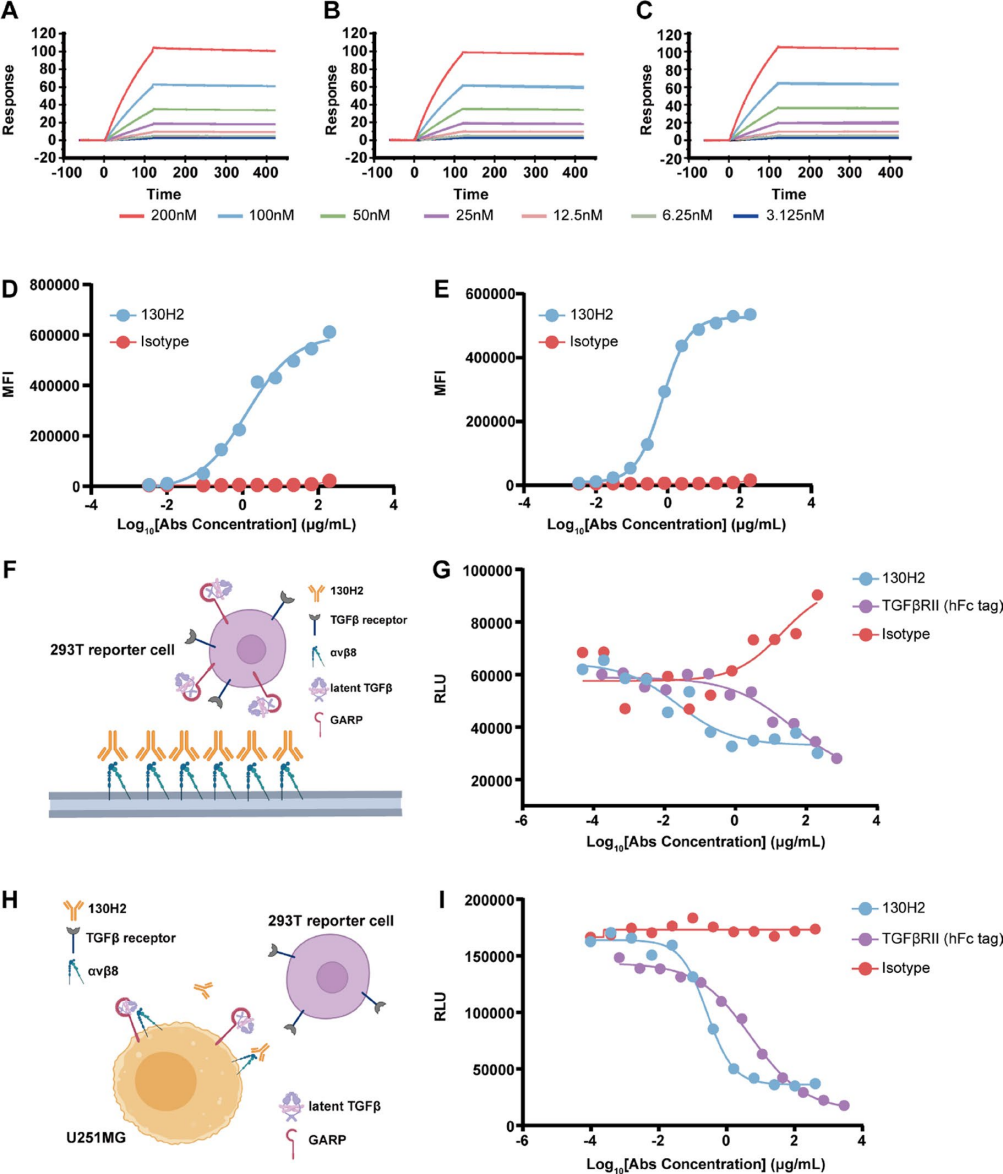
In vitro characterization of the anti-αvβ8 antibody 130H2. (**A**-**C**) 130H2 demonstrates robust binding capacity to serially diluted (concentration ranging from 200 to 3.125 nM) human αvβ8 protein (**A**), cynomolgus αvβ8 protein (**B**), and mouse αvβ8 protein (**C**) as determined by Biacore system. **(D-E)** Flow cytometry-based binding curves of threefold diluted 130H2 starting from 200 nM to native U251MG cells (**D**) and OVCAR3 cells (**E**) detected by APC labeled secondary antibody. The curves were fitted using sigmoidal four parameter logistic (4PL) model. **(F)** Schematic illustration of the protein-cell interaction system used in Fig. 2G. The TGF-β luciferase reporter HEK293T cell line, co-transfected with GARP, latent TGF-β1, and TGF-βRII, was used as effector cells. **(G)** The blocking activity of 130H2 was evaluated by reporter-based assay. Concentration dependent inhibition curves of 130H2 compared with TGFβRII trap were generated by incubating them with αvβ8 and HEK293T/GARP/latent-TGFβ1/TGFβRII-luciferase cells for 5 h and detected by luciferase substrate. **(H)** Schematic illustration of the cell-cell interaction system used in Fig. 2I. U251MG cells, which natively express both integrin αvβ8 and latent TGF-β1, were used as target cells. The TGF-β luciferase reporter HEK293T cell line, co-transfected with GARP, latent TGF-β1, and TGF-βRII, was used as effector cells. **(I)** The blocking activity of 130H2 was determined by U251MG-effector cell interaction system. Human HEK293T/GARP/latent-TGFβ1/TGFβRII-luciferase cells were incubated with U251MG cells (E: T = 2: 1) for 5 h in the presence of serial dilutions of 130H2 or TGFβRII trap and the blocking reaction was detected by measuring luminescence units

To further investigate the expression and function of αvβ8, we generated a specific monoclonal antibody (mAb), designated 130H2, using hybridoma technology. This antibody was subsequently humanized through complementarity-determining region (CDR) grafting. The binding affinity of mAb 130H2 to human, cynomolgus, and mouse αvβ8 was assessed using Biacore, demonstrating high affinity with dissociation constants (KD) of 2.848 nM, 1.897 nM, 1.292 nM for each species, respectively (Fig. 2A, B, C). The highly binding specificity of 130H2 was confirmed by testing against the other integrin family proteins using another αvβ8 antibody C6D4 as a control (supplemental Fig. 2). The ability of mAb 130H2 to bind native αvβ8 was evaluated using the U251MG human malignant glioblastoma cell line, which is known to express integrins natively [38]. Flow cytometry (FACS) results showed that 130H2 actively binds to surface αvβ8, with a half-maximal effective concentration (EC_50_) of 1.32 nM (Fig. 2D). This binding was further confirmed using another αvβ8-positive cell line, OVCAR3, with an EC_50_ of 0.71 nM (Fig. 2E).

To evaluate the efficacy of 130H2 to block TGF-β activation, we utilized a TGF-β luciferase reporter HEK293T cell line co-transfected with GARP, latent TGF-β1, and the TGF-βRII [30, 39]. The TGF-β trap (TGF-βRII-hFc) was used as a positive control. As depicted in Fig. 2F, the 130H2 antibody effectively blocks the interaction between integrin αvβ8 and latent TGF-β, thereby inhibiting the release of downstream reporter gene signals. The 130H2 antibody inhibited luciferase gene expression with a low half-maximal inhibitory concentration (IC_50_) of 0.02 nM, indicating its effectiveness in blocking TGF-β activation. Notably, 130H2 demonstrated superior efficacy compared to the TGF-β trap, which had an IC_50_ of 37.15 nM, particularly at low concentrations (Fig. 2G). The blocking function of 130H2 was further validated in a cell-cell interaction system using U251MG cells, which express both integrin αvβ8 and latent TGF-β to activate TGF-β in the luciferase reporter cells (Fig. 2H). The 130H2 antibody potently inhibited TGF-β activation in U251MG cells, with an IC_50_ of 0.26 nM, showcasing greater efficacy than the TGF-β trap, which had an IC_50_ of 5.24 nM, especially at low concentrations (Fig. 2I).

In summary, the antibody 130H2 exhibits high affinity and specificity for human αvβ8 and effectively inhibits TGF-β activation.

### Cryo-EM analysis demonstrates antibody specificity

**Fig. 3:**
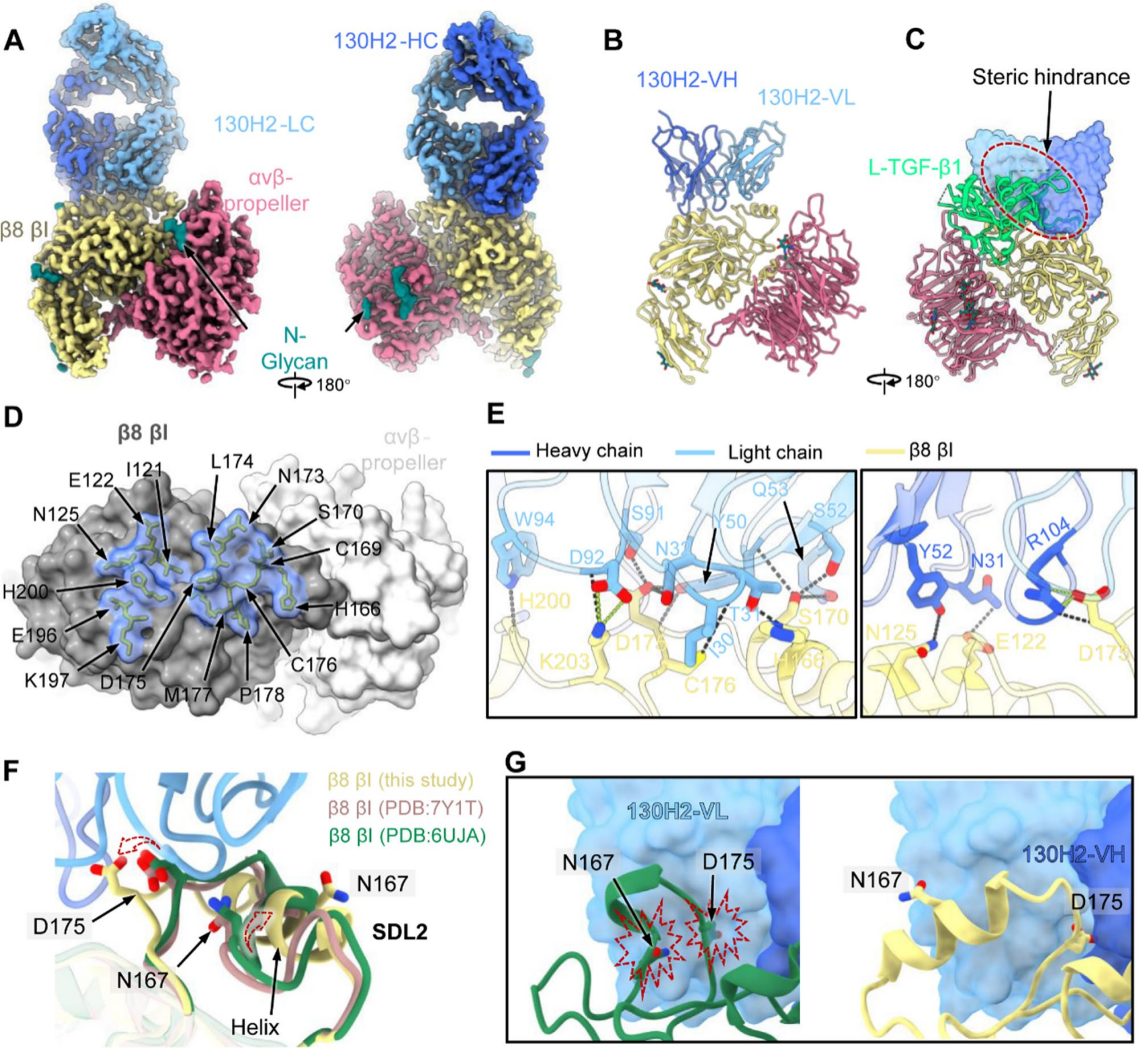
Cryo-EM structure of human integrin αvβ8 ectodomain in complex with 130H2-Fab. **(A)** The domain-colored density maps of αvβ8 in complex with 130H2-Fab. Light blue and dark blue represent the light and heavy chain of 130H2-Fab, yellow represents β8 βI domain, and pink represents αvβ-propeller, respectively. **(B)** The established atomic model of αvβ8 bound with 130H2-Fv, represented by cartoon style. **(C)** Superimposition of αvβ8-130H2 and αvβ8-L-TGF-β1 (PDB ID: 6UJA) illustrates the steric hindrance between 130H2 and L-TGF-β1 (green). **(D)** The footprint of 130H2 on αvβ8. The αvβ8 is shown as a gray surface representation, with residues involved in 130H2 interaction depicted as a transparent surface and sticks. (**E)** The interaction network between the αvβ8 and 130H2. The heavy chain and light chain mediate a network of hydrogen bonds (black dashed lines) and a salt bridge (green dashed lines). The side chains of key residues involved in hydrogen bonds or salt bridges are labeled and shown as sticks. **(F)** The binding of 130H2 induces a conformational change in the SDL2 of the βI domain on αvβ8. Key residues were pointed by arrows. Yellow SDL2 represent structure obtained in this study, purple and green SDL2 are from PDB ID 7Y1T and 6UJA, respectively. **(G)** The triggered conformational change (right) is to prevent the original steric clashes (left) between the key residues N167 and D175, with 130H2

To investigate the molecular mechanism of mAb 130H2, we employed cryo-EM single particle analysis for the αvβ8 ectodomain in complex with the antigen-binding fragment (Fab) of 130H2. The resulting αvβ8:130H2 complex yielded a high-resolution cryo-EM structure at 2.88 Å resolution (Fig. 3A, supplemental Fig. 3 and supplemental Table 2), allowing for the construction of the atomic model of partial headpiece of the heterodimeric αvβ8, including the αv β-propeller and β8 (βI and hybrid) subunit, as well as the bound variable regions of 130H2 Fab (Fig. 3B). Superimposition of the αvβ8:130H2 and αvβ8: L-TGF-β1 structures revealed that 130H2 could block the L-TGF-β1 binding to αvβ8 via steric hindrance (Fig. 3C), explaining its ability to inhibit TGF-β activation.

Unlike other reported mAbs, such as C6D4, which mimic the binding mode of L-TGF-β1 by interacting with both αv and β8 subunits (supplemental Fig. 4) [40], 130H2 exclusively contacts the β8 βI subunit of the heterodimeric αvβ8, utilizing both its heavy and light chains. The footprint of 130H2 covers an area of approximately 1,000 Å² at the interface, comprising 15 contacting residues: I121, E122, N125, H166, C169, S170, N173, L174, D175, M177, P178, E196, K197 and H200 (Fig. 3D). These residues include the specificity-determining loop 2 (SDL2), which is critical for TGF-β binding. Within the interaction network at the interface, the light chain forms the primary interaction with β8 βI, contributing 12 hydrogen bonds and 2 salt bridges (Fig. 3E). In contrast, the heavy chain forms 5 hydrogen bonds and 2 salt bridges (Fig. 3E).

Interestingly, compared to L-TGF-β1-bound, the binding of 130H2 induces a dramatic conformational change in the SDL2 loop of βI (Fig. 3F). This loop deflects outward and transitions into a more stable and rigid helical conformation (Fig. 3F). This conformational change is driven by the representative residues, N167 and D175, which influence the binding of 130H2 and trigger an adaptive local conformational rearrangement to avoid steric clashes (Fig. 3G). Consequently, this conformational change enables 130H2 to compete with L-TGF-β1 for binding, further disrupting the SDL2 conformation required for L-TGF-β1 binding, thus contributing to the highly inhibitory action of 130H2 on TGF-β activation. Collectively, our structural insights unveil the unique binding pattern of 130H2 targeting integrin αvβ8, providing a mechanistic understanding of how 130H2 inhibits TGF-β activation through both steric hindrance and conformational changes.

### Anti-αvβ8 antibody inhibits tumor growth and reverses immunosuppression by enhancing immune cell infiltration

**Fig. 4:**
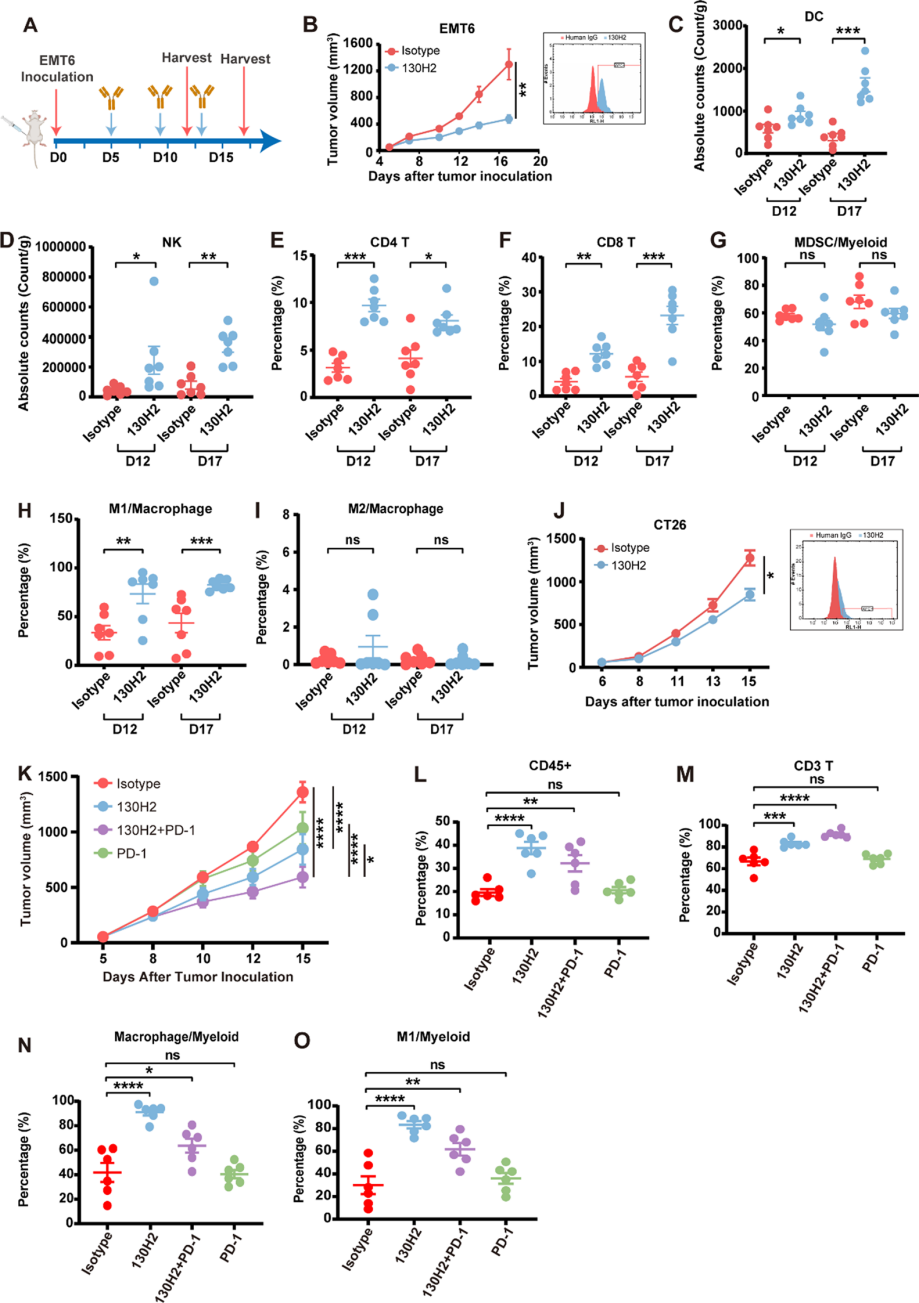
130H2 inhibited tumor growth by increasing immune cells infiltration. **(A)** Experimental schematics of the workflow for in vivo efficacy and TILs analysis. **(B** and **J)** BALB/c mice (*n* = 7) bearing EMT6 cells (B) and CT26 cells (**J**) were intraperitoneally treated with 10 mg/kg 130H2 or isotype antibody every four days on day 5, 9, 13 post inoculation, three times in total. Significance, *P < 0.05 by Student’s t-test, **P < 0.01 by Student’s t-test. The expression of αvβ8 in the EMT6 cell line and CT26 cell line before inoculation, visualized by staining with 130H2 antibody, is shown in the right. **(C-I)** BALB/c mice (*n* = 7 every time in each group) were sacrificed on day 12 and 17 after tumor inoculation and EMT6 tumors were dissociated to analyze tumor-infiltrating immune cells using FACS. Absolute number of cells per gram tumor tissue were calculated for populations of DCs (**C**) and NK cells (**D**). Proportion of CD4 + T cell (**E**) and CD8 + T cell (**F**) populations were determined by CD3 + CD4 + CD8- and CD3 + CD4-CD8 + markers. Myeloid cell populations including MDSC/myeloid ratio (**G**), M1/macrophage (**H**), and M2/macrophage (**I**) were evaluated. *P < 0.05, **P < 0.01, ***P < 0.001, ****P < 0.0001 were analyzed by Student’s t-test. *P* < 0.05 means statistical significance. **(K)** BALB/c mice (*n* = 6) bearing EMT6 cells were intraperitoneally administrated with the following treatments: 10 mg/kg isotype-hIgG1(day 5, 12) plus 10 mg/kg isotype-rat IgG2a (day 5, 9, 13), 10 mg/kg 130H2 (day 5, 12) plus 10 mg/kg isotype-rat IgG2a (day 5, 9, 13), 10 mg/kg PD-1 antibody (day 5, 9, 13) plus 10 mg/kg isotype-hIgG1 (day 5, 12), 10 mg/kg 130H2 (day 5, 12) plus 10 mg/kg PD-1 antibody (day 5, 9, 13). *P* < 0.0001 was indicated as ****, *P* < 0.05 as *, and statistical significance was determined by two-way ANOVA with Tukey’s multiple comparisons test. **(L-O)** BALB/c mice (*n* = 6 in each group) were sacrificed on day 15 post-tumor inoculation, and EMT6 tumors were dissociated for analysis of tumor-infiltrating immune cells using FACS. The proportions of CD45 + cell, CD3 + T cell, macrophages, and M1 macrophages were quantified. Statistical significance was determined by one-way ANOVA with Dunnett’s multiple comparisons. Data in figures B-O are presented as mean ± SEM

We conducted an evaluation on the efficacy of 130H2 in suppressing tumor growth, utilizing an immune-excluded mouse syngeneic model, EMT6, and performed an analysis on tumor-infiltrating lymphocytes (TILs) as depicted in Fig. 4A. EMT6 was primarily distinguished by significantly low levels of αvβ8 expression as illustrated in Fig. 4B. Initiation of antibody treatment was enacted when the tumor volume arrived at an approximation of 100 mm³. Notably, the application of 130H2 as targeted monotherapy resulted in a significant suppression of tumor growth, achieving a TGI value of 63% with a dosage of 10 mg/kg (Student’s t-test, *P* < 0.01, Fig. 4B). To elucidate the mechanism of tumor inhibition by 130H2 monotherapy, we examined TILs. EMT6 tumors were harvested on day 12 post-inoculation (isotype group tumor volume ~ 500 mm³) and day 17 post-inoculation (isotype group tumor volume ~ 1200 mm³) following 130H2 antibody treatment, and TILs were analyzed via FACS (Fig. 4C-I). Compared to the control group, 130H2 treatment significantly increased the percentage of infiltrated innate and adaptive immune cells on both day 12 and day 17. Notably, there were substantial increases in tumor-infiltrating DCs, NK cells, CD4 + T cells, and CD8 + T cells (Fig. 4C-F). The absolute counts of CD4 + and CD8 + T cells per gram of tumor weight showed a remarkable rise with 130H2 antibody treatment (supplemental Fig. 5A-B). Furthermore, the percentage of M1 macrophages increased significantly, while no significant changes were observed in myeloid-derived suppressor cells (MDSCs) and M2 macrophages (Fig. 4G-I). The absolute cell count of M1 macrophages per gram of tumor weight was also determined (supplemental Fig. 5C). Collectively, these results indicate that 130H2 treatment enhances the infiltration of DCs, NK cells, CD4 + T cells, CD8 + T, and M1 macrophages into the tumor microenvironment, effectively reversing its immunosuppressive phenotype.

In a parallel study, the efficacy of 130H2 was evaluated in the CT26 syngeneic mouse model, which exhibits undetectable levels of αvβ8 expression (Fig. 4J). Despite the lack of detectable αvβ8 expression, 130H2 treatment continued to suppress tumor growth significantly (Student’s t-test, *P* < 0.05; Fig. 4J). Analysis of TILs revealed a similar pattern to that observed in the EMT6 model. Specifically, CD3 + T cell infiltration was significantly increased (Supplemental Fig. 5G). However, the macrophage populations infiltrating CT26 tumors differed slightly from those in EMT6 tumors. In CT26, M1 macrophages were scarce, and the predominant macrophage populations consisted of M1&M2 intermediate-state and M2 phenotypes. Following 130H2 treatment, the M2 macrophage population was significantly reduced, while the M1&M2 intermediate-state population increased. These findings suggest that 130H2 treatment shifted the macrophage population toward a more M1-like phenotype.

The EMT6 model is known to be insensitive to PD-1 treatment due to limited T cell infiltration within the tumor [41]. Since anti-αvβ8 antibody treatment can increase immune cell infiltration, the synergistic effect of anti-αvβ8 antibody and anti-PD-1 antibody was investigated (Fig. 4K). 10 mg/kg of 130H2 monotherapy displayed a potent anti-tumor effect with a TGI of 38% (*P* < 0.0001). Notably, the combination treatment group demonstrated significantly enhanced tumor suppression, yielding a TGI value of 56% (two-way ANOVA with multiple comparisons test, *P* < 0.0001; Fig. 4K). The combination therapy was markedly more effective than either PD-1 monotherapy (*P* < 0.0001) or 130H2 monotherapy (*P* < 0.05).

Further analysis of TILs revealed that the combination treatment group exhibited increased immune cell infiltration and CD3 + T cell populations, similar to the effects observed with 130H2 monotherapy (Fig. 4L-O). In contrast, PD-1 monotherapy alone had no significant impact on immune cell infiltration (Fig. 4L, M). Since anti-PD-1 therapy enhances T cell activity, the combination group benefits from a synergistic anti-tumor effect by simultaneously increasing immune cell infiltration (via 130H2) and activating T cells (via PD-1 blockade). Interestingly, similar to 130H2 monotherapy, the combination treatment also led to an increase in the M1 macrophage population (Fig. 4N, O).

In summary, multiple efficacy studies accompanied by TIL analyses consistently demonstrated that the 130H2 antibody significantly enhances immune cell infiltration into tumors, with a notable increase in the proportion of M1 macrophages. Targeting αvβ8 with the 130H2 antibody effectively reprograms tumor-infiltrating immune cells, leading to potent anti-tumor effects.

### Regulation of macrophage polarization by anti-αvβ8 antibody

**Fig. 5:**
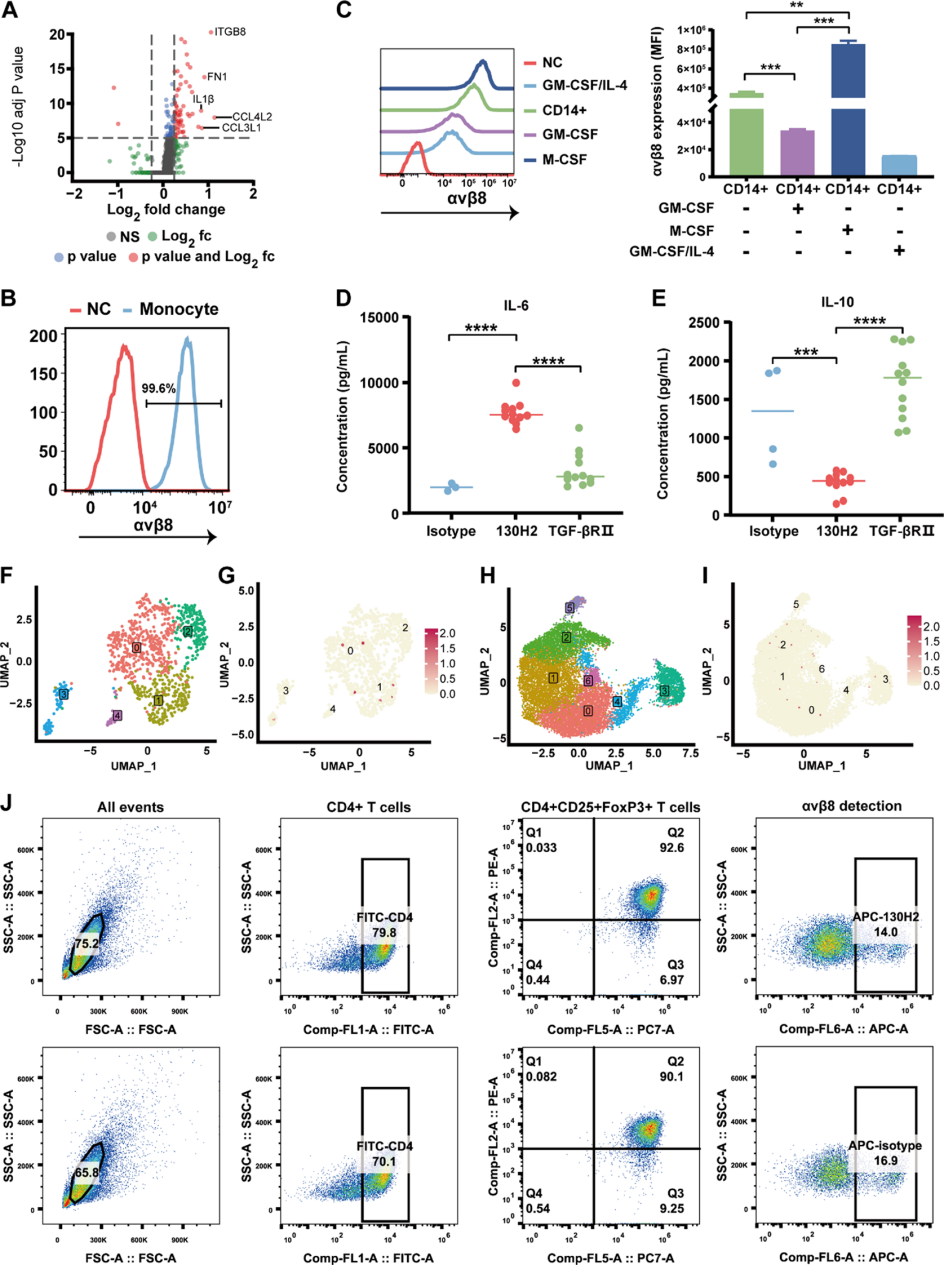
130H2 regulates macrophage polarization. **(A)** Volcano plot illustrating differentially expressed genes between ITGB8 + and ITGB8 − macrophages. Differential gene expression was calculated using the FindMarkers function with the Wilcoxon Rank Sum test in the Seurat package. The filtering criteria were set as: adjusted p-value (P-adj) < 0.05 and log_2_(fold change) ≥ 0.25. **(B)** The αvβ8 expression on monocyte isolated from PBMC by CD14 positive selection kit was detected by flow cytometry. NC represents the absence of staining. **(C)** Monocytes were differentiated to M1, M2 macrophages, and DCs stimulated by GM-CSF, M-CSF or GM-CSF/IL-4, respectively. The expression of αvβ8 on these myeloid cells was detected by FACS (left). Mean fluorescence intensity (MFI) values were quantified and presented as bar graphs (right), with statistical significance indicated. **(D-E)** CD14 + monocytes were stimulated by M-CSF for 4 days, followed by adding 130H2, TGFβRII trap or an isotype control for an additional 3 days. Cytokine levels of IL-6 and IL-10 were quantified by HTRF technology. **(F-I)** Feature plots showing the distribution of Treg subpopulations (**F** and **H**) and *ITGB8* expression in Treg cells (**G**, **I**) derived from Figure 1. **(J)** Treg cells isolated from human PBMCs were stimulated with a CD3/CD28 activator and IL-2. CD4 + CD25 + FoxP3 + Tregs were detected using APC-labeled 130H2 or an APC-labeled isotype control. Representative data are shown. The data in figure C, D, E was shown as mean ± SEM. **P < 0.005, ***P < 0.0005, ****P < 0.0001 was determined by Student’s t-test

In previous research, most scientists have focused on the role of αvβ8 in tumor-infiltrated human Tregs [39, 42]. However, using scRNA-seq analysis, we identified high expression of the β8-encoding gene *ITGB8* in tumor-infiltrating macrophages. Further analysis revealed distinct gene expression profiles between ITGB8 + and ITGB8- macrophages. Notably, ITGB8 + macrophages exhibited elevated levels of pro-tumorigenic markers associated with M2-like tumor-associated macrophages, including *FN1* [43], *IL1β* [44], *CCL4L2* [45], and *CCL3L1* [46] (Fig. 5A). To confirm αvβ8 expression in myeloid cells, CD14 + monocytes were isolated from human PBMCs and stained with an anti-αvβ8 antibody. Flow cytometry analysis revealed high expression levels of αvβ8 in the CD14 + monocytes (Fig. 5B). Subsequently, these monocytes were cultured in the presence of GM-CSF, M-CSF, or GM-CSF/IL-4 to induce M1 or M2 macrophage phenotypes or DCs, respectively. The M1, M2, and DC phenotypes were characterized using specific markers (supplemental Fig. 6). The expression of αvβ8 was detected in all myeloid cells, with the ranking of expression levels being DC < M1 < monocyte < M2 (Fig. 5C). Mean fluorescence intensity (MFI) values for the different treatment groups, as shown in Fig. 5C (left), were quantified and represented as bar graphs in Fig. 5C (right), with statistical significance clearly indicated. Notably, the expression of αvβ8 was significantly upregulated in the M2 phenotype, while it was downregulated in the M1 phenotype (Fig. 5C right), indicating that αvβ8 expression is associated with macrophage polarization.

Previous reports indicated the absence of αvβ8 expression on murine monocytes isolated from the spleen [6]. However, findings from the TILs study, which demonstrated a significant increase in mouse macrophage infiltration following αvβ8 antibody treatment, prompted an investigation into the expression of αvβ8 on murine myeloid cells using FACS. Murine monocytes derived from PBMCs, spleen, and bone marrow were examined. Surprisingly, high αvβ8 expression was detected in both Ly6C^high^ and Ly6C^low^ monocyte populations, regardless of the source of the monocyte cells (supplemental Fig. 7A-C). Additionally, high levels of αvβ8 expression were observed on murine DCs (supplemental Fig. 7D) and macrophages (supplemental Fig. 7E), indicating a widespread presence of αvβ8 on these immune cell populations in mice.

To investigate the impact of the anti-αvβ8 antibody on macrophage polarization, monocytes were incubated with αvβ8 antibody, and cytokine release was assessed. Treatment with the antibody 130H2 resulted in increased secretion of the pro-inflammatory cytokine IL-6 compared to the isotype control (Fig. 5D, *P* < 0.0001). While TGF-βRII served as a positive control, treatment with 130H2 notably surpassed TGF-βRII treatment in enhancing IL-6 secretion. Furthermore, levels of the anti-inflammatory cytokine IL-10 were measured, revealing a significant decrease in IL-10 secretion following 130H2 treatment (Fig. 5E, *P* < 0.001). These findings demonstrate that the antibody 130H2 can effectively modulate macrophage polarization towards a more pro-inflammatory M1 phenotype.

Previous studies reported that αvβ8 is expressed in human Tregs and exerts a suppressive effect on anti-tumor immunity [42, 47]. To further investigate this, Tregs from the data sources of Fig. 1B and F were collected and reclustered to assess *ITGB8* gene expression (Fig. 5F-I). Surprisingly, there was minimal *ITGB8* gene expression observed in Tregs (Fig. 5G, I). To confirm this observation, we analyzed *ITGB8* expression in tumor-infiltrating Tregs across various tumor tissues. Consistently, our findings revealed that *ITGB8* expression in Tregs was consistently low (supplemental Fig. 8). To further validate this finding, Treg cells were isolated from human PBMCs from five donors and stimulated with a CD3/CD28 activator and IL-2. While robust expression of CD4 + CD25 + FoxP3 + was observed in the stimulated Tregs, αvβ8 expression remained undetectable. Representative data are shown in Fig. 5J. Previous reports on αvβ8 expression in Tregs have not been validated at the protein level through antibody staining or Western blot analysis. It is proposed that the expression of αvβ8 in Tregs is likely undetectable, and any potential effects on Tregs may be minimal. In summary, αvβ8 is primarily expressed on monocytes and macrophages, and the anti-αvβ8 antibody may regulate macrophage polarization by promoting a pro-inflammatory phenotype.

### Enhanced efficacy of anti-αvβ8 in tumors with high αvβ8 expression

**Fig. 6:**
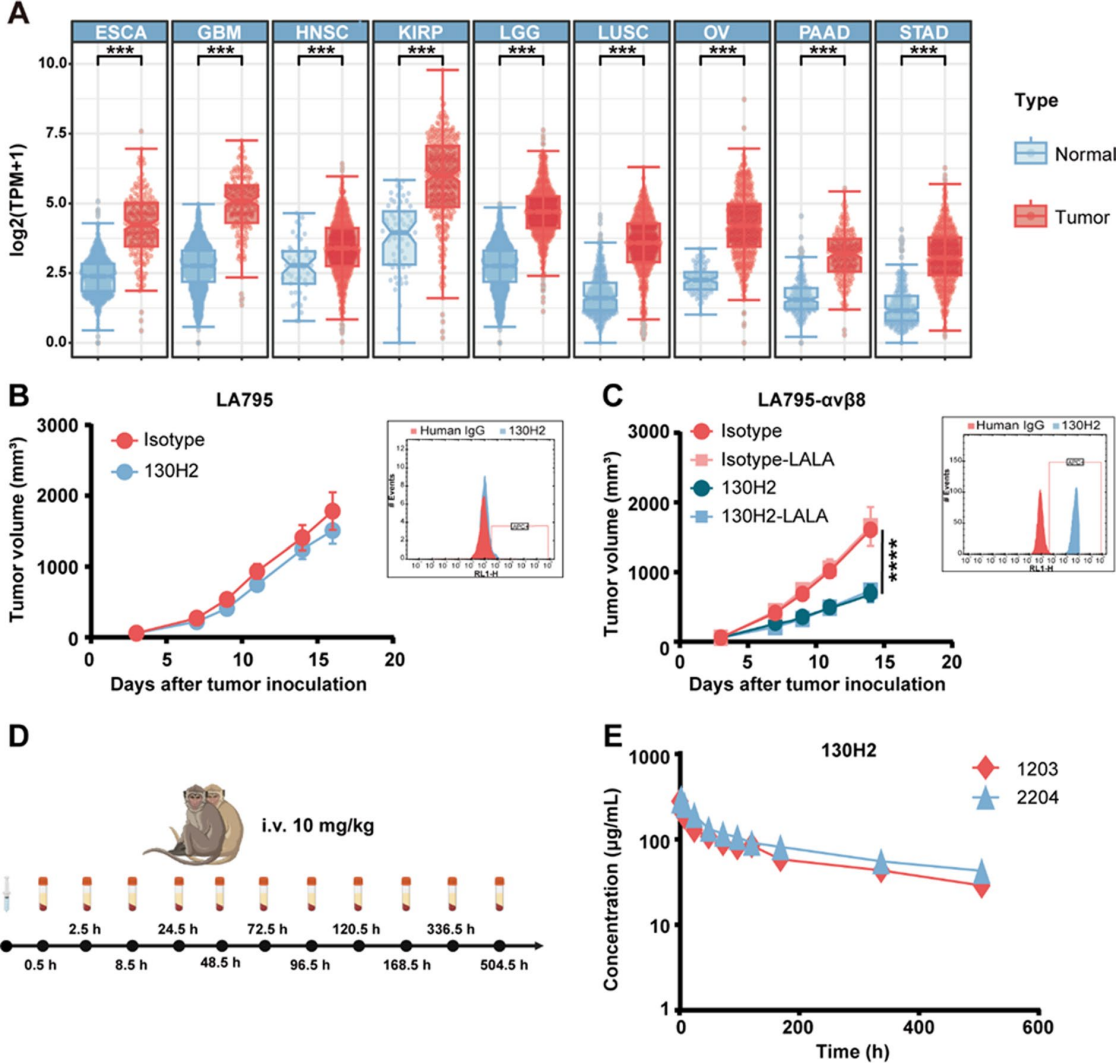
High expression of αvβ8 significantly enhanced the in vivo efficacy of 130H2. **(A)** mRNA expression of αvβ8 was compared across 9 distinct tumors (red) and corresponding normal tissues (blue) from the TCGA and GTEx projects. **(B)** LA795 cells bearing Kunming mice (*n* = 7) were intravenously treated with 10 mg/kg 130H2 or isotype control every four days for three times. Tumor volume was measured and shown as mean ± SEM. The expression of αvβ8 on LA795 was detected by FACS and the histogram was embedded beside the tumor growth curve. **(C)** Kunming mice (*n* = 7 per group) bearing human αvβ8 overexpressed LA795 cells were intravenously treated with 5 mg/kg 130H2, 130H2-LALA, isotype control or isotype-LALA every four days for three times. The overexpression of αvβ8 on LA795-hαvβ8 is shown at the right of tumor growth curve. Tumor volume was recorded and shown as mean ± SEM. *P* value of 130H2 verse isotype was determined by two-way ANOVA, ****P < 0.0001. **(D)** Timeline graphical representation of PK and safety evaluation in two cynomolgus monkeys, which were administrated 10 mg/kg 130H2 via intravenous infusion. **(E)** The concentration of 130H2 in serum was measured by ELISA

We conducted an analysis of mRNA expression in various human tumors, revealing a significantly higher expression of the *ITGB8* gene in numerous tumors compared to normal tissues (Fig. 6A). Due to the challenge of identifying mouse syngeneic models with high αvβ8 expression, we opted to overexpress αvβ8 in mouse lung adenocarcinoma LA795 cells. Subsequently, we treated mice transplanted with LA795 naïve cells and LA794-αvβ8 cells with the anti-αvβ8 antibody 130H2.

Interestingly, while only a minor tumor inhibition effect was observed in the LA795 models, a significant anti-tumor effect was noted in the LA795-αvβ8 models (Fig. 6B, C, *P* < 0.0001). To rule out cytotoxicity related to Fc function, an Fc silent LALA mutation was introduced into the Fc region of the 130H2 antibody. The efficacy of both the original 130H2 antibody and the mutated 130H2-LALA antibody was compared in the LA795-αvβ8 mouse models. Surprisingly, no difference was observed between these two groups, indicating that the anti-tumor effect was not mediated by the antibody’s Fc function. Our findings suggest that the observed anti-tumor effect was primarily due to the blocking function of TGF-β rather than the antibody’s Fc region. Furthermore, tumors expressing αvβ8 were found to be more sensitive to the TGF-β blocking effect, highlighting the potential significance of αvβ8 expression in tumor response to such therapies.

The pharmacokinetic (PK) and safety evaluation of the 130H2 antibody was conducted in non-human primates (NHPs), specifically two cynomolgus monkeys, one male and one female. The antibody 130H2 was administered at a dosage of 10 mg/kg via intravenous infusion, with continuous monitoring over a 3-week period. Hematology, blood biochemistry, and PK parameters were assessed (Fig. 6D). No abnormal observations related to the antibody administration were noted in any of the animals during the experiment. The pharmacokinetic analysis of 130H2 in serum, performed using WinNonlin 8.3 software, revealed a relatively long average half-life of 338 h and a maximum concentration (C_max_) of 291 µg/mL (Fig. 6E).

In conclusion, the 130H2 antibody demonstrated high potency in αvβ8 expression tumors, along with favorable pharmacokinetic properties and safety profiles.

## Discussion

TGF-β is a crucial immunosuppressive factor within the TME, exerting its effects through various pathways. However, the systemic blockade of TGF-β as a therapeutic strategy for tumor progression has been challenging due to its highly context-dependent functions. Through scRNA-seq, we identified that integrin αvβ8, a known activator of TGF-β, is highly expressed in both tumor cells and tumor-infiltrating macrophages. In response, we developed a specific antibody targeting integrin αvβ8, named 130H2, to inhibit TGF-β signaling within the TME.A previous study described another anti-αvβ8 antibody, C6D4, which was found to interact with both the β8 subunit and the αv head domain, indicating potential nonspecific binding with other αv-containing proteins. In contrast, our structural analysis of the 130H2 antibody complexed with latent TGF-β (L-TGF-β) demonstrated that 130H2 exclusively binds to the β8 subunit, underscoring its specificity.

Expression of integrin αvβ8 was observed in epithelial cells [48, 49], fibroblasts [50], neurons, and glial cells [51], as well as in DCs [12]. Recently, expression of αvβ8 in tumor microenvironment was reported but whether αvβ expressed in tumor cells or in Treg cell are still in debate [13, 40, 52–55]. In our study, scRNA-seq revealed that *ITGB8* is highly expressed in epithelial cells or epithelial-derived tumor cells, as well as in tumor-infiltrating macrophages. Further investigation revealed that *ITGB8*-positive macrophages exhibit high expression of functional genes associated with M2 macrophages, such as *FN1*, *IL1β*, and *CCL4L2*, suggesting that these macrophages exhibit an M2-like phenotype. Notably, we did not observe significant αvβ8 expression in Treg cells infiltrating various tumor types.

To further explore αvβ8 expression in Tregs, we isolated Treg cells from PBMCs and activated them using CD3/CD28 and IL-2, followed by FACS analysis. The results indicated minimal αvβ8 expression in these cells, leading us to speculate that αvβ8 likely plays a negligible role in Treg function. In contrast, high expression of αvβ8 was detected by FACS in CD14 + monocytes isolated from human PBMCs and in vitro-induced macrophages. Interestingly, when CD14 + monocytes were incubated with M-CSF to induce anti-inflammatory M2-like macrophages, the expression of αvβ8 increased dramatically. Conversely, treatment with GM-CSF to promote M1-like pro-inflammatory macrophages led to a marked decrease in αvβ8 expression. These results suggest that αvβ8 may be directly involved in macrophage polarization. Furthermore, incubation with an αvβ8-specific antibody significantly altered macrophage function, as evidenced by a dramatic increase in pro-inflammatory IL-6 secretion and a concomitant decrease in anti-inflammatory IL-10 secretion. This observation confirms that blocking αvβ8 can shift macrophage polarization toward a more pro-inflammatory M1-like phenotype.

A previous study concluded that αvβ8 is not expressed on tumor cells but is instead restricted to tumor-infiltrating Treg cells [42]. This conclusion was drawn from the absence of detectable αvβ8 expression in disaggregated mouse tumor cells, including those from αvβ8-expressing cell lines such as CCK168 and TRAMP-C2. While their findings are valid within the scope of their experimental design, alternative methodologies might yield additional insights. For instance, immunohistochemical (IHC) staining of human tumor tissues has consistently demonstrated significant αvβ8 expression localized to tumor cells [56], suggesting potential differences in expression patterns between species or experimental conditions. The authors also employed FACS-based sorting of immune cells from murine tumors followed by RT-PCR to assess αvβ8 expression. However, in our attempts to replicate similar analyses, we encountered technical challenges due to the low abundance of non-T cell immune populations in murine tumors, which made it difficult to generate reliable data. In comparison, approaches such as single-cell transcriptomics using fresh human tumor tissues appear to offer more robust and comprehensive insights. These considerations highlight the importance of incorporating diverse models and methods to achieve a more complete understanding of αvβ8 expression and its role in tumor biology.

In preclinical models, treatment with 130H2 significantly inhibited tumor growth in the immune-excluded EMT6 model. TILs analysis demonstrated that 130H2 monotherapy enhanced immune cell infiltration and increased the population of pro-inflammatory M1 macrophages. Further investigation confirmed that αvβ8-blocking antibodies promote macrophage polarization towards the M1 phenotype, a finding also validated in the αvβ8-deficient CT26 model. In the CT26 tumors, baseline macrophage populations were predominantly M1&M2 intermediate and M2 phenotypes, with limited M1 macrophages. Following 130H2 treatment, the M2 population was significantly reduced, accompanied by an increase in M1&M2 intermediate populations and enhanced overall immune cell infiltration, indicating a favorable shift in the tumor immune microenvironment. The combination of αvβ8-blocking antibodies with PD-1 inhibitors produced a synergistic antitumor effect. This dual treatment significantly increased immune cell infiltration, including CD3 + T cells and M1 macrophages, similar to the changes observed with 130H2 monotherapy. Given that anti-PD-1 therapy enhances T cell activity, the combination therapy exerts a synergistic anti-tumor effect by concurrently facilitating immune cell infiltration through 130H2 and activating T cells via PD-1 blockade.

To validate our findings and better understand the therapeutic potential of αvβ8 integrin blockade in combination with immune checkpoint inhibitors, further testing across a broader range of mouse models is essential. Given the spatial and temporal context dependency of TGF-β signaling, evaluating αvβ8 integrin inhibition in tumor models representing diverse biological and immune environments is crucial. Models derived from different organ sites, such as melanoma, lung cancer, and colorectal cancer, will help determine whether αvβ8 blockade exerts organ-specific effects on the tumor microenvironment. Additionally, comparing primary versus metastatic tumors, as well as orthotopic versus subcutaneous models, could provide insights into how tumor progression and the native tumor environment influence therapeutic outcomes.

Importantly, further studies should explore tumor models reflecting the three major immune phenotypes—immune-inflamed, immune-excluded, and immune-desert [57].These phenotypes, also observed in human solid tumors, are characterized by distinct immune microenvironments that influence their responsiveness to immunotherapies. Immune-inflamed tumors, with abundant T cell infiltration, typically respond well to PD-1/PD-L1 inhibitors. In contrast, immune-excluded tumors restrict T cells to the tumor periphery, often due to elevated TGF-β signaling, while immune-desert tumors exhibit minimal immune cell presence and are largely unresponsive to immunotherapy. Our study utilized EMT6 (immune-excluded) and CT26 (moderately inflamed) mouse models to demonstrate that αvβ8 integrin blockade enhances the efficacy of anti-PD-1/PD-L1 therapy. Expanding to other models, such as inflamed (e.g., melanoma) and immune-desert (e.g., neuroendocrine) tumors, would allow systematic evaluation of αvβ8 inhibition across diverse immune contexts, providing critical insights into the generalizability and clinical relevance of these findings.

Among these phenotypes, TGF-β signaling is particularly associated with the immune-excluded phenotype, where it suppresses T cell infiltration and anti-tumor immunity. Tumors with high TGF-β signaling gene signatures often correlate with poor prognosis across various cancer types [58]. In our study, PD-1 antibody monotherapy was ineffective in the immune-excluded EMT6 model. However, combining the αvβ8 antibody, which inhibits TGF-β activation, resulted in significant tumor inhibition, suggesting that simultaneous blockade of TGF-β signaling and PD-1/PD-L1 can overcome the immunosuppressive barriers of the immune-excluded tumor microenvironment. This combination strategy enables T cells to infiltrate the tumor and restore anti-tumor immune responses, highlighting its potential to improve the efficacy of PD-1 inhibitors in resistant immune-excluded tumors. Further clinical investigation of this combination therapy in immune-excluded tumors, as well as its potential in immune-desert tumors, could pave the way for more effective, personalized cancer immunotherapies.

## Electronic supplementary material

Below is the link to the electronic supplementary material.

Supplementary Material 1

## Data Availability

No datasets were generated or analysed during the current study.

## Abbreviations

TGF-β: Transforming Growth Factorβ
TME: Tumor Microenvironment
NK: Natural Killer Cells
LAP: Latency-Associated Peptide
DCs: Dendritic Cells
Tregs: Regulatory T Cells
scRNA-seq: single-cell RNA sequencing
ESCA: Esophageal Cancer
LIHC: Liver Hepatocellular Carcinoma
LSCC: Lung Squamous Cell Carcinoma
NPC: Nasopharyngeal Cancer
NSCLC: Non-Small Cell Lung Cancer
OSCC: Oral Squamous Cell Carcinoma
KIRC: Kidney Renal Clear Cell Carcinoma
TISCH2: Tumor Immune Single-cell Hub 2
ATCC: American Type Culture Collection
MEM: Minimum Essential Medium
FBS: Fetal Bovine Serum
RLU: Relative Luminescence Units
CTF: Contrast Transfer Function
PBMCs: Peripheral Blood Mononuclear Cells
M-CSF: Macrophage Colony-Stimulating Factor
GM-CSF: Granulocyte-Macrophage Colony-Stimulating Factor
IL-6: Interleukin-6
IL-10: Interleukin-10
HTRF: Homogeneous Time-Resolved Fluorescence
ELISA: Enzyme-Linked Immunosorbent Assay
TGI: Tumor Growth Inhibition
UMAP: Uniform Manifold Approximation and Projection
mAb: Monoclonal Antibody
Fab: Antigen-Binding Fragment
CDR: Complementarity-Determining Region
KD: Dissociation Constants
FACS: Flow Cytometry
EC_50_: Half-Maximal Effective Concentration
IC_50_: Half-Maximal Inhibitory Concentration
SDL2: Specificity-Determining Loop 2
TILs: Tumor-Infiltrating Lymphocytes
MDSCs: Myeloid-Derived Suppressor Cells
PK: Pharmacokinetics
NHPs: Non-Human Primates
C_max_: Maximum Concentration
