# Supplementary material for: Specifically blocking αvβ8-mediated TGF-β signaling to reverse immunosuppression by modulating macrophage polarization
Source: J Exp Clin Cancer Res. 2025 Jan 2;44:1. doi: 10.1186/s13046-024-03250-1 (PMC11697059; doi:10.1186/s13046-024-03250-1)
Supplement: Supplementary file 1 — Supplementary Material 1 [file 13046_2024_3250_MOESM1_ESM.docx]

**SUPPLEMENTARY MATERIALS**

**Supplementary methods**

**Specificity characterization of 130H2**

96-well ELISA plate (Costar, USA) was coated with 1 μg/mL (100 μL/well) human-αvβ1、human-αvβ3、human-αvβ5、human-αvβ6 overnight at 4 ℃. Next day, the plate was washed three times with washing buffer (0.1% PBST) and blocked by blocking buffer (0.1% PBST containing 2% BSA) for 2 h at 37 ℃. After discarding the blocker, threefold serially diluted 130H2 or control antibodies starting from 100 μg/mL were added to the plate for 1 h incubation at 37 ℃. After another three times washing, the plate was incubated with 1: 5000 diluted Horseradish peroxidase (HRP) labeled goat anti-human IgG Fcγ secondary antibody for 1 h at 37 ℃. The binding assay was developed by TMB and stopped by 1 N HCl. The absorbance was read at 450 nm with the SpectraMax M5e Microplate Reader.

**Murine monocytes preparation**

Blood was collected from BALB/c mice via the tail vein and stored in anticoagulant tubes. Following euthanasia, the spleen and femurs were carefully isolated according to standard protocols. Murine PBMCs were separated from the blood by density gradient centrifugation using Ficoll-Paque solution (Cytiva). The spleen was ground into a single-cell suspension, and red blood cells were lysed using RBC lysis buffer (Invitrogen, USA). The femurs were cut at both ends and flushed with phosphate-buffered saline (PBS) to collect bone marrow cells. Finally, PBMCs, spleen cells, and bone marrow cells were labeled with CD11b (BioLegend, USA), Ly6G (BioLegend), and Ly6C (BioLegend) markers, as well as an αvβ8 antibody, to analyze αvβ8 expression.

**BMDCs and BMDMs preparation**

Mononuclear cells isolated from bone marrow were seeded at a density of 1×10^6^ cells/mL in RPMI 1640 medium containing 10% FBS. To generate bone marrow-derived dendritic cells (BMDCs), the culture medium was supplemented with 20 ng/mL murine GM-CSF (Peprotech, USA) and 20 ng/mL murine interleukin-4 (mIL-4; Peprotech). Additionally, 50 ng/mL murine M-CSF (Peprotech) was added to induce the differentiation of bone marrow-derived macrophages (BMDMs). After seven days, both dendritic cells and macrophages were collected to assess the expression of αvβ8.

**Supplementary Figures**

**
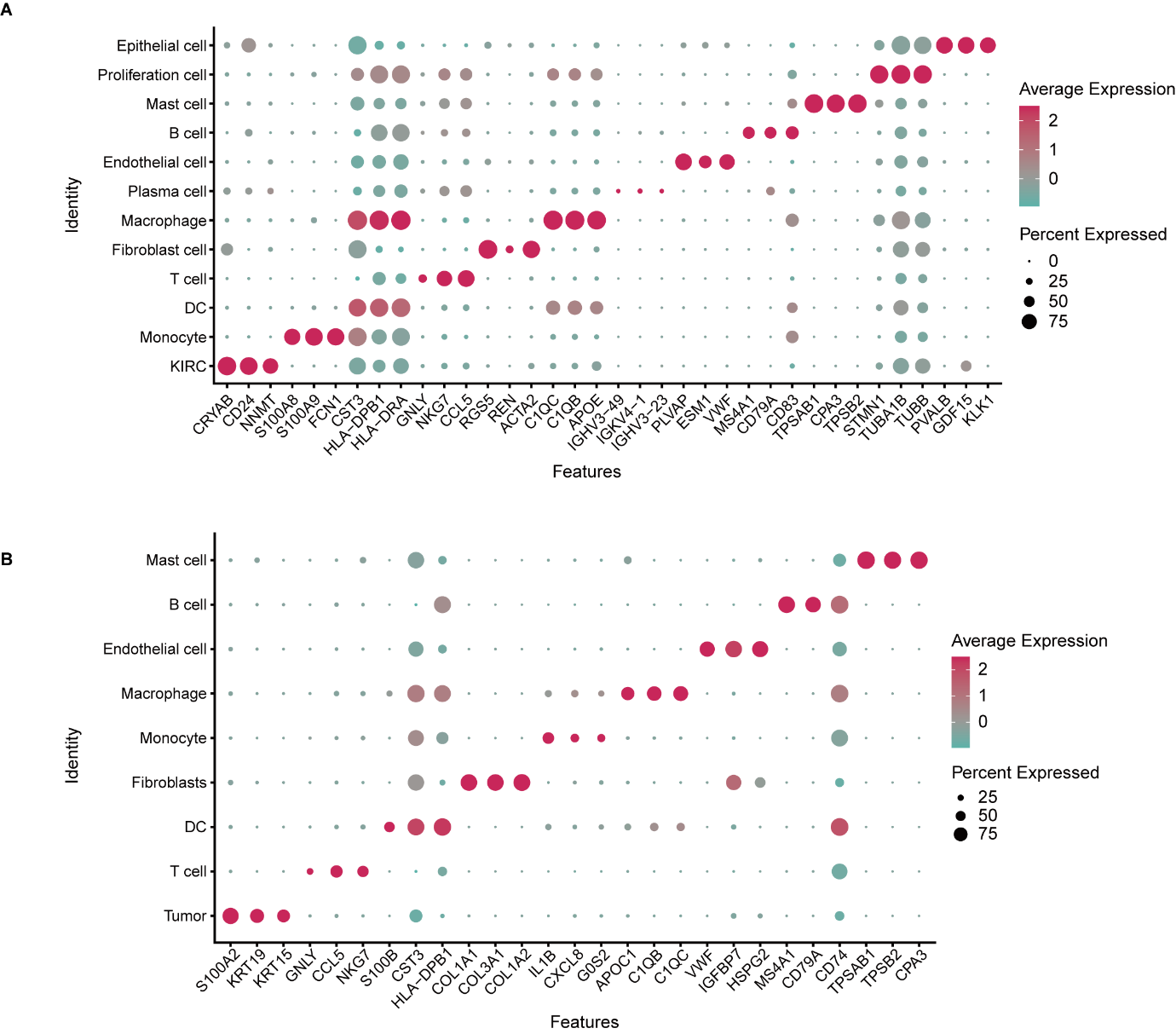
**

**Supplemental Fig. 1. Marker genes for the KIRC and HNSC cell clusters. (A-B)** The marker genes of 12 cell types in KIRC (A), and marker genes of 9 cell types in HNSCC (B) as described in Fig. 1.


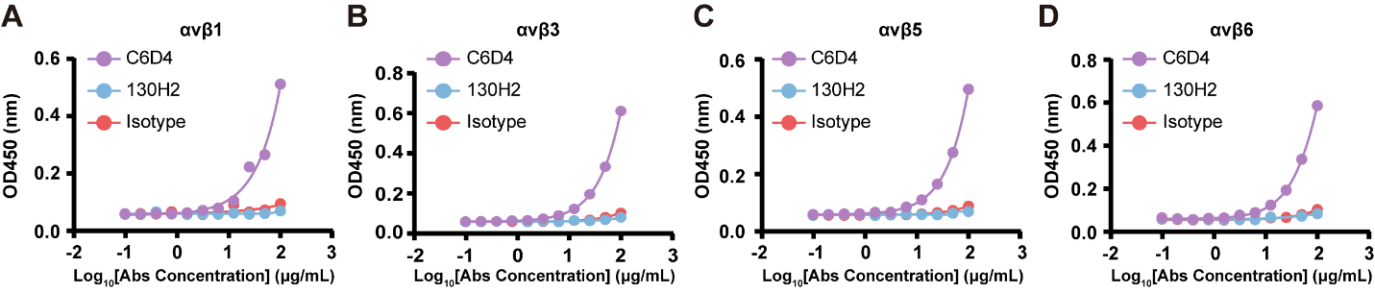


**Supplemental Fig. 2. High binding specificity of anti-αvβ8.** **(A-D)** Human αvβ1 (A), αvβ3 (B), αvβ5 (C), αvβ6 (D) in the integrin family was coated onto the ELISA plate and detected by 130H2 and HRP labeled secondary antibody. Antibody C6D4, which can bind to the αv subunit, was used as a control.

**
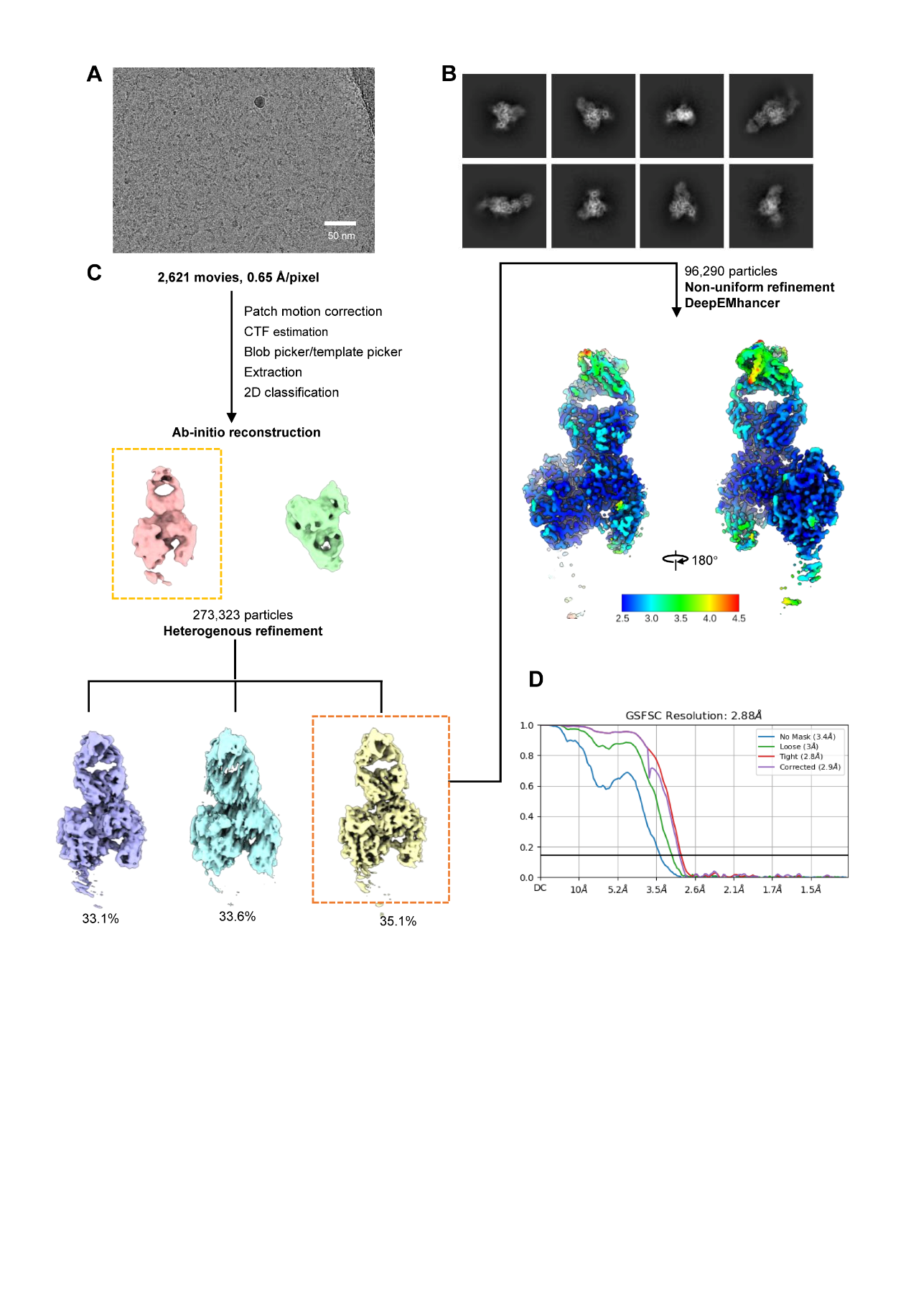
**

**Supplemental Fig. 3. Single-particle cryo-EM data processing workflow for αvβ8:130H2.** **(A)** Representative electron micrograph of frozen 130H2-Fab/αvβ8 complex recorded using 300 kV Titan Krios G4 transmission electron microscope with Gatan K3 camera (scale bar: 50 nm). **(B)** Representative 2D class averages. **(C)** The detailed reconstruction workflow for αvβ8:130H2. **(D)** FSC curves for the reconstruction is shown.

**
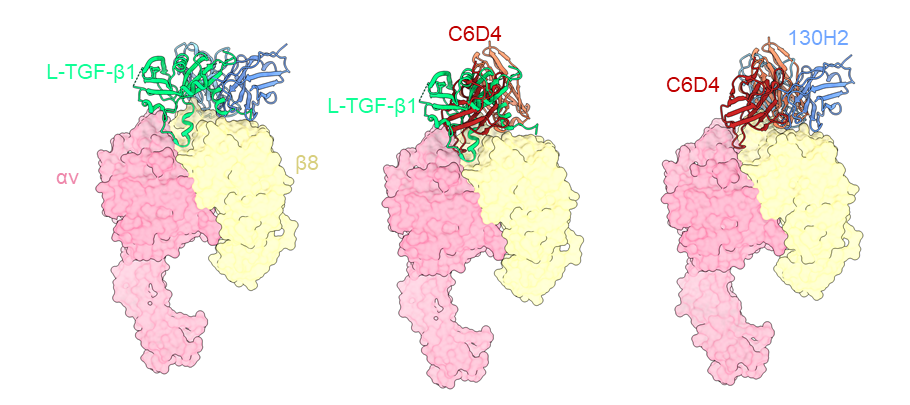
**

**Supplemental Fig. 4. Comparison of the binding modes of 130H2, C6D4 and L-TGF-β1.** The binding mode of 130H2 to αvβ8 compared with L-TGF-β1 (left), C6D4 to αvβ8 compared with L-TGF-β1 (middle) and comparison of 130H2 with C6D4 (right).

**
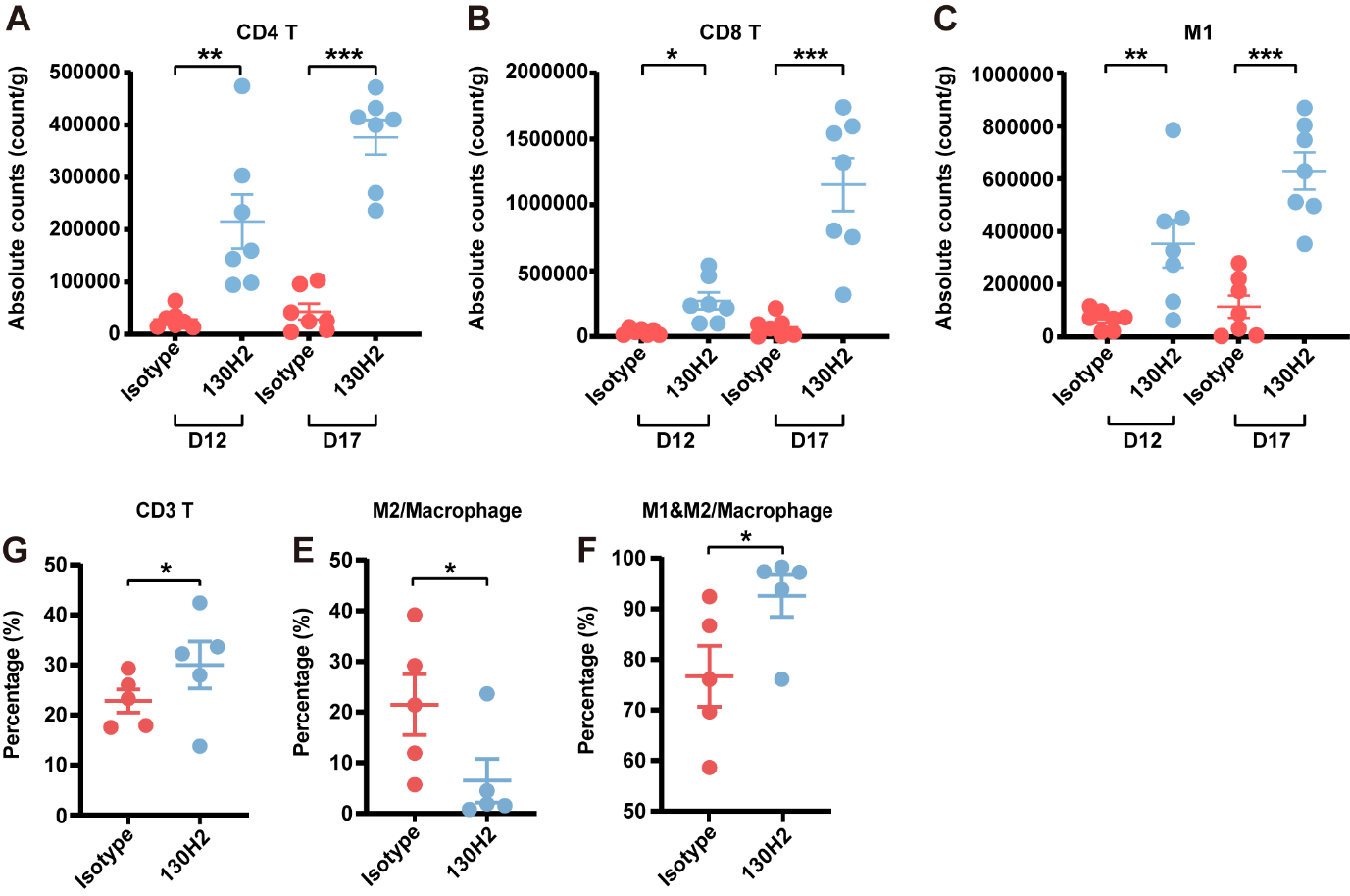
**

**Supplemental Fig. 5. Absolute counts of immune cells after 130H2 monotherapy.** **(A-C)** BALB/c mice were administrated with 10 mg/kg 130H2 or isotype antibody on day 5, 9, 13 and sacrificed on days 12 and 17. EMT6 tumors were dissociated to analyze tumor-infiltrating immune cells using FACS. Absolute number of cells per gram tumor tissue were calculated for populations of CD4 + T cells (A), CD8 + T cells (B) and M1 macrophages (C). ***P* < 0.01, ****P* < 0.001, *****P* < 0.0001 were analyzed by Student’s t-test. **(G-F)** CT26 tumors were dissociated to analyze tumor-infiltrating immune cells using FACS. Proportion of CD3 + T cells (G), M2 macrophages (E) and M1&M2 macrophages (F). **P* < 0.05, were analyzed by Student’s t-test.


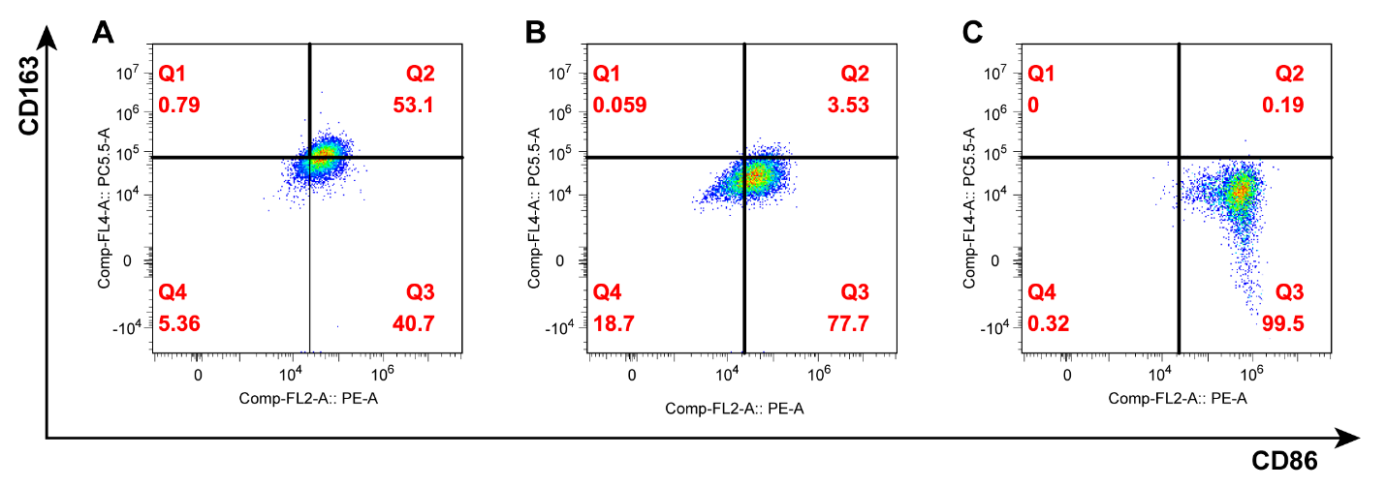


Supplemental Fig. 6. Macrophage differentiation characterization. (A-C) Monocytes were stimulated by M-CSF, GM-CSF, or MG-CSF plus IL-4 to differentiate into M2-like macrophages (A), M1-like macrophages (B), or DCs (C), which were distinguished by CD86 and CD163 markers.

**
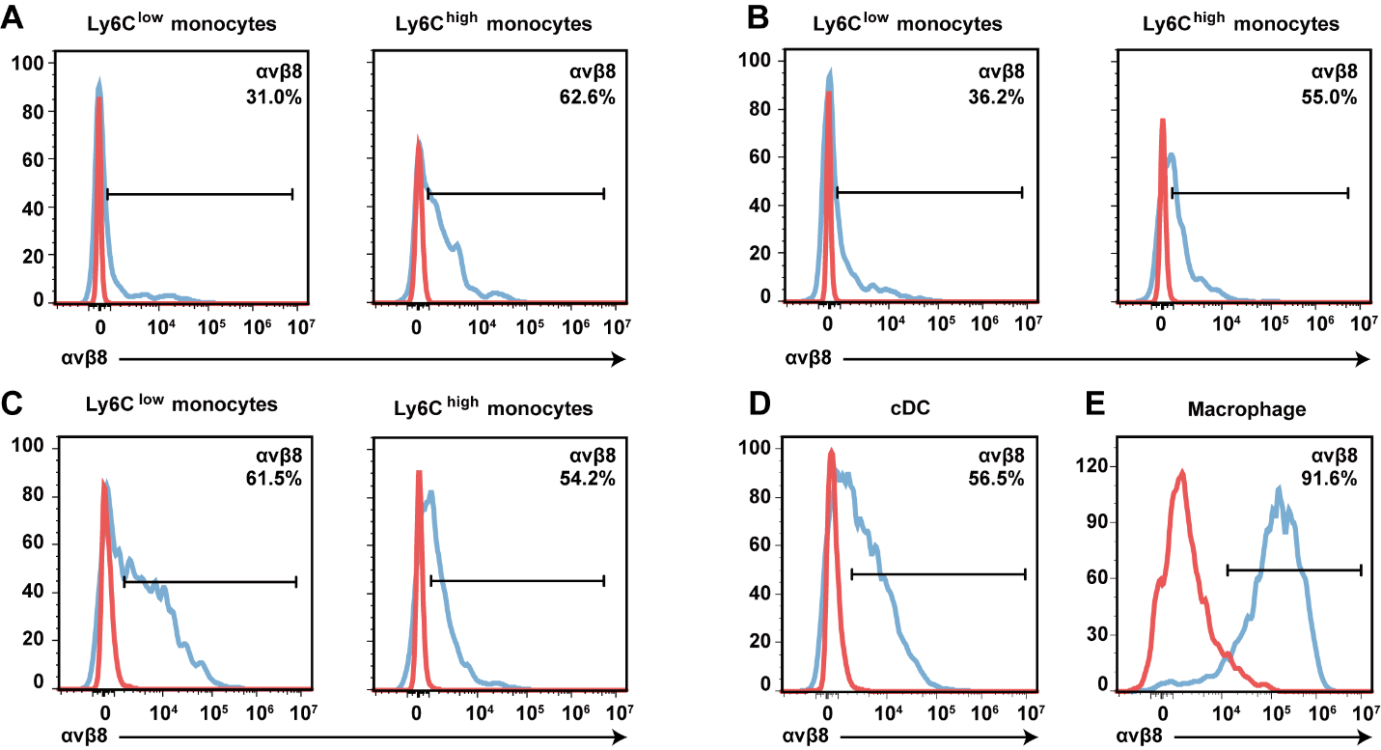
**

**Supplemental Fig. 7. The expression of αvβ8 in murine immune cells. (A-C)** Murine monocytes isolated from PBMCs (A), spleen (B), and bone marrow (C) were stained by Ly6C to distinguish Ly6C^low^ and Ly6C^high^ monocytes and the expression of αvβ8 was analyzed by FACS. **(D-E)** Mononuclear cells derived from bone marrow were stimulated by murine GM-CSF and IL-4 to differentiate cDCs (D) or murine M-CSF to obtain macrophages (E), followed by evaluating the expression of αvβ8 by FACS.

**
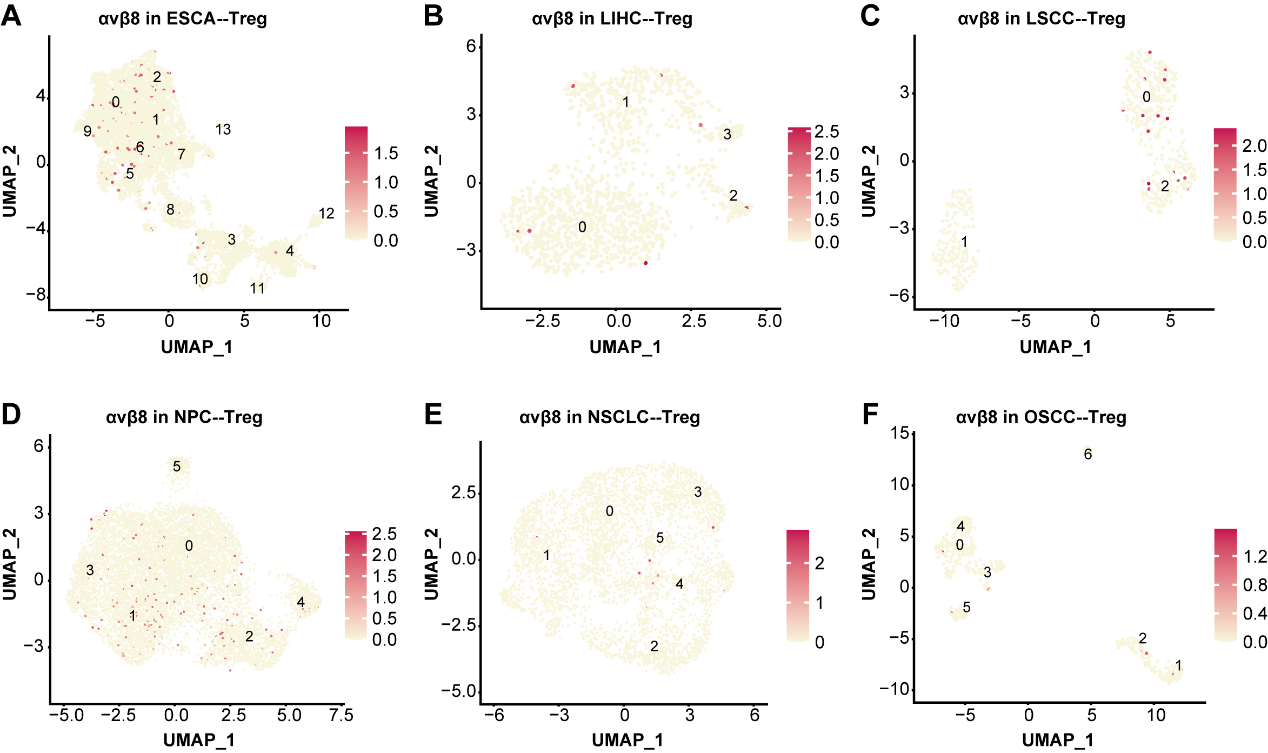
**

**Supplemental Fig. 8. Expression of αvβ8 in Treg cells across six different tumor types. (A-F)** scRNA-seq datasets were obtained from the following sources: ESCA-GSE160269 (A), LIHC-GSE166635 (B), LSCC-GSE150321 (C), NPC-GSE162025 (D), NSCLC-E-MTAB-6149 (E), OSCC-GSE172577 (F). Treg cell subpopulations were identified and extracted after data acquisition, followed by dimensionality reduction and clustering. The expression of αvβ8 was visualized using feature plots. Subpopulation markers were determined based on the original research papers or the TISCH database.

**Supplementary Tables**

**Supplemental Table 1. Samples from patients with KIRC.**

| **ID** | **Age** | | **Cancer type** | **Clinical Stage** | **Sample site** | **The Eighth edition AJCC cancer stage** |
| --- | --- | --- | --- | --- | --- | --- |
| KIRC-1 | 67 | kidney renal clear cell carcinoma | | I | Right kidney | 1 |
| KIRC-2 | 66 | kidney renal clear cell carcinoma | | II | Right kidney | 2 |
| KIRC-3 | 57 | kidney renal clear cell carcinoma | | II | Left kidney | 2 |
| KIRC-4 | 55 | kidney renal clear cell carcinoma | | II | Left kidney | 1 |
| KIRC-5 | 51 | kidney renal clear cell carcinoma | | II | Right kidney | 1 |
| KIRC-6 | 69 | kidney renal clear cell carcinoma | | II | Right kidney | 1 |
| KIRC-7 | 72 | kidney renal clear cell carcinoma | | I | Left kidney | 2 |
| KIRC-8 | 59 | kidney renal clear cell carcinoma | | II | Left kidney | 2 |
| KIRC-9 | 63 | kidney renal clear cell carcinoma | | I-II | Left kidney | 1 |
| KIRC-10 | 52 | kidney renal clear cell carcinoma | | II | Left kidney | 1 |

**Supplemental Table 2. Cryo-EM data collection, refinement, and validation statistics of the αvβ8:130H2.**

|  | αvβ8:130H2 complex |
| --- | --- |
| **Data Collection and processing** |  |
| Microscope | Krios G4 |
| Camera | K3 |
| Voltage (kV) | 300 |
| Electron exposure dose (e^−^/Å^2^) | 50 |
| Defocus range (μm) | 0.8–1.4 |
| Pixel size (Å) | 0.65 |
| Micrographs (used) | 2,621 |
| Final particle images (nos.) | 96,290 |
| Symmetry imposed | C1 |
| Map resolution (Å) | 2.88 |
| FSC threshold | 0.143 |
| Map sharpening B factor (Å^2^) | -69.3 |
| **Validation** |  |
| MolProbity score | 1.61 |
| Poor rotamers (%) | 0.26 |
| Clashscore | 5.06 |
| RMS (bonds) | 0.0108 |
| RMS (angles) | 1.32 |
| *Ramachandran plot* |  |
| Favored (%) | 95.10 |
| Allowed (%) | 4.90 |
| Disallowed (%) | 0 |
